# High-contrast, fast chemical imaging by coherent Raman scattering using a self-synchronized two-colour fibre laser

**DOI:** 10.1038/s41377-020-0259-2

**Published:** 2020-02-24

**Authors:** Cihang Kong, Christian Pilger, Henning Hachmeister, Xiaoming Wei, Tom H. Cheung, Cora S. W. Lai, Nikki P. Lee, Kevin. K. Tsia, Kenneth K. Y. Wong, Thomas Huser

**Affiliations:** 1 Department of Electrical and Electronic Engineering, The University of Hong Kong, Pokfulam Road, Hong Kong, China; 2 Biomolecular Photonics, Department of Physics, University of Bielefeld, Universitätsstr, 25, 33615 Bielefeld, Germany; 3 Division of Life Science, The Hong Kong University of Science and Technology, Hong Kong, China; 4 Department of Physiology, Li Ka Shing Faculty of Medicine, The University of Hong Kong, Pokfulam, Hong Kong, China; 5 Department of Surgery, The University of Hong Kong, Pokfulam Road, Hong Kong, China; 6 Present Address: Division of Engineering and Applied Science, California Institute of Technology, 1200 East California Boulevard, Pasadena, CA 91125 USA

**Keywords:** Fibre lasers, Confocal microscopy

## Abstract

Coherent Raman scattering (CRS) microscopy is widely recognized as a powerful tool for tackling biomedical problems based on its chemically specific label-free contrast, high spatial and spectral resolution, and high sensitivity. However, the clinical translation of CRS imaging technologies has long been hindered by traditional solid-state lasers with environmentally sensitive operations and large footprints. Ultrafast fibre lasers can potentially overcome these shortcomings but have not yet been fully exploited for CRS imaging, as previous implementations have suffered from high intensity noise, a narrow tuning range and low power, resulting in low image qualities and slow imaging speeds. Here, we present a novel high-power self-synchronized two-colour pulsed fibre laser that achieves excellent performance in terms of intensity stability (improved by 50 dB), timing jitter (24.3 fs), average power fluctuation (<0.5%), modulation depth (>20 dB) and pulse width variation (<1.8%) over an extended wavenumber range (2700–3550 cm^−1^). The versatility of the laser source enables, for the first time, high-contrast, fast CRS imaging without complicated noise reduction via balanced detection schemes. These capabilities are demonstrated in this work by imaging a wide range of species such as living human cells and mouse arterial tissues and performing multimodal nonlinear imaging of mouse tail, kidney and brain tissue sections by utilizing second-harmonic generation and two-photon excited fluorescence, which provides multiple optical contrast mechanisms simultaneously and maximizes the gathered information content for biological visualization and medical diagnosis. This work also establishes a general scenario for remodelling existing lasers into synchronized two-colour lasers and thus promotes a wider popularization and application of CRS imaging technologies.

## Raman-scattering microscopy: Fiber laser reduces the bulk

An extremely stable fiber laser makes the high-resolution imaging technique of coherent Raman scattering (CRS) microscopy more practical for use in hospitals. CRS employs two pulsed laser beams to probe human tissues, providing structural and chemical information without the need for chemical labelling. However, current CRS systems require bulky laser systems that are impractical for clinical use. Now, Thomas Huser at Bielefeld University in Germany, with coworkers in Hong Kong (Kenneth K. Y. Wong) and the USA (Xiaoming Wei), have demonstrated CRS imaging using a compact pulsed fiber laser that provides excellent stability over a wide frequency range. Their system produced fast, high contrast images of living human cells and mouse tissues including the kidney and brain. Moreover, the fiber-based laser beam could easily be connected to other optical imaging systems such as endoscopes, to acquire images inside the body.

## Introduction

Optical imaging techniques have long served as important tools for biomedical applications^1^. Among these techniques, coherent Raman scattering (CRS), which encompasses coherent anti-Stokes Raman scattering (CARS)^2^, stimulated Raman scattering (SRS)^3–6^, and other nonlinear optical processes^7–13^, have been successfully applied to tackle biomedical problems by providing chemically specific, label-free contrast, high spectral and spatial resolution, and high sensitivity. CRS microscopy, in particular, enables minimally invasive and continuous live imaging of biomolecular samples^14,15^ where fluorescent molecules cannot be applied or their use might interfere with biological applications due to the size, weight and toxicity of fluorophores; in addition, the bleaching of fluorophores and the phototoxicity of the excitation light often prevent long-term imaging or disturb the natural behaviour of biological samples. The clinical translation of CRS imaging technologies, however, has largely been stalled by the high cost and bulkiness of conventional laser sources. Standard solid-state lasers, typically Ti:sapphire lasers and synchronously pumped optical parametric oscillators (OPOs) using free-space optics, usually have to be mounted on vibration-isolated optical tables, which creates a major technical obstacle for clinical applications. This requirement is even more important for CRS imaging that requires two coherent pulsed laser sources with sufficient power and low-intensity noise at two different wavelengths covering a suitable range of probed wavenumbers, which must also be tightly synchronized and have matched pulse widths for generating an optimal CRS signal. Finally, both pulsed laser sources have to be temporally and spatially overlapped on sub-picosecond time and nanometre length scales, respectively, while the stabilities in both domains should also be ensured with high reliability.

To this end, various two-colour pulsed fibre lasers have recently been proposed and demonstrated for CRS imaging, including fibre-based Ti:sapphire hybrid sources^16,17^ and all-fibre lasers based on processes such as active synchronization^18,19^, parametric wavelength conversion^20–23^, soliton self-frequency shift^24–29^ and supercontinuum (SC) light generation^30–35^. These approaches, however, have specific limitations, either high cost, low power or high intensity noise—the major challenge for directly demodulating a weak SRS signal (on the order of 10^−4^ – 10^−6^)^4^; see Supplementary Material 1. As a result, thus far, there is still no entirely fibre-based coherent two-colour pulsed laser source available that provides sufficiently high-power and low-intensity noise for fast SRS imaging without balanced detection—a technology for suppressing laser noise that requires delicate adjustments of balanced receivers to subtract the exceeding intensity noise; however, it is difficult to maintain the performance of balanced detection across a wide spectral range for dynamic samples, and balanced detection can also increase the complexity and cost of an imaging system.

Recent developments in advanced fibre optics have created new opportunities for generating high-quality ultrafast laser pulses and have expanded their application potential^36–39^, including (1) heavy doping of active fibres, enabling high output power per unit length up to hundreds of milliwatts from a centimetre-long active fibre^40,41^; (2) the design of double-cladding fibres (DCFs)^42,43^, which allows efficient cladding pumping based on cost-effective multimode fibre-coupled pump laser diodes (MMFPLDs) and creates brightness orders of magnitude higher than the core pumping scheme^44^; (3) fibre chirped-pulse amplification (FCPA) technology^45^, which further boosts the energy and thus the peak power of ultrashort laser pulses by eliminating the detrimental pulse distortion that mainly arises from fibre nonlinearities; and (4) finally, the fabrication of multifunctional hybrid fibre components that reduce the overall complexity of ultrafast fibre lasers and enhance their robustness^46,47^. By leveraging these advanced fibre optic technologies, we present a novel high-power, low-noise, self-synchronized two-colour pulsed fibre laser system utilizing coherent wavelength generation (CWG) through cross-phase modulation (XPM)^48^. With this system, we demonstrate high-quality CARS and SRS imaging, and other nonlinear imaging modalities without the need for balanced detection, which is otherwise required to cancel laser noise in SRS imaging^32,35^, enabling high scan speeds while keeping the complexity of the detection setup low.

## Results

**Fig. 1:**
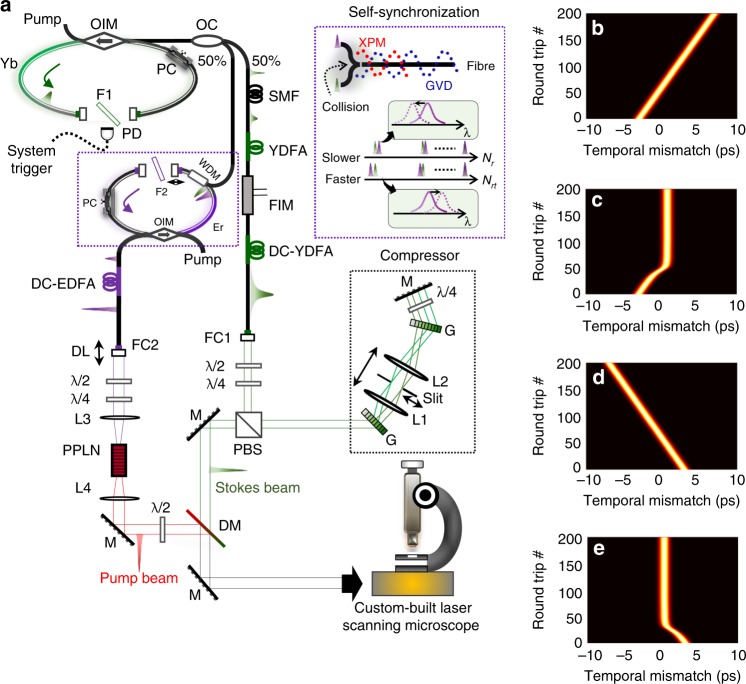
Self-synchronized two-colour pulsed fibre laser for multimodal nonlinear optical microscopy. **a** Schematic diagram of the two-colour pulsed fibre laser. A passively mode-locked fibre laser at 1.0 µm generates a fs pulse train at a repetition rate of 80 MHz. The output is split into two branches by a fibre optic coupler (OC) to generate the Stokes and pump beams. After spatially combining Stokes and pump beams by a dichroic mirror (DM) and temporally overlapping the beams by a delay line (DL), the two-colour laser beams are launched into a custom-built laser scanning microscope. A detailed description of the experimental setup is provided in Supplementary Material 2. DC-EDFA double-cladding erbium/ytterbium-doped fibre amplifier, DC-YDFA double-cladding ytterbium-doped fibre amplifier, Er erbium-doped fibre, F filter, FC fibre collimator, FIM fibre-coupled intensity modulator, G grating, GVD group velocity dispersion, L lens, M mirror, *N*_*rt*_ round-trip number, OIM fibre-based optically integrated module, PBS polarizing beam splitter, PC polarization controller, PD photodiode, PPLN periodically poled lithium niobate crystal, SMF single-mode fibre, WDM wavelength-division multiplexing coupler, XPM cross-phase modulation, Yb ytterbium-doped fibre, YDFA ytterbium-doped fibre amplifier, λ/2 half-wave plate, λ/4 quarter-wave plate. **b**–**e** Numerical simulations of the timing of 1.0 ps pulses at 1.5 µm without (**b**, **d**) and with (**c**, **e**) self-synchronization. The simulation assumes that the 1.5 µm pulses have an initial round-trip time mismatch of 50 fs that is either slower (**b**, **c**) or faster (**d**, **e**). Further details are provided in Supplementary Material 3

Different from prior realizations of synchronized pulse generation typically seeded by noise^20–35^, here, the synchronization between the two-colour pulsed laser beams is based on CWG with a passive self-stabilization scheme. Figure 1a depicts the configuration of the self-synchronized two-colour pulsed fibre laser source; further details are provided in Supplementary Material 2. The laser system starts from a passively mode-locked fs fibre laser at a wavelength of 1.0 µm, named the master laser. The laser cavity consists of a short piece of ytterbium-doped fibre (Yb), a drop-in polarization controller (PC) and a fibre-based optically integrated module (OIM) that provides polarization-sensitive isolation, pump/signal multiplexing and signal extraction. Passive mode-locking is realized by nonlinear polarization rotation (NPR), a reliable technique for generating self-starting and stable fs pulses in the all-normal dispersion regime^49^. The output pulse train has an average power of ~20 mW and a fundamental repetition rate (FRR) of 80 MHz. Such a high FRR can, in particular, enable the fast modulation of pulse intensities necessary for high-speed SRS imaging. The output is split into two parts by utilizing a 50:50 fibre optic coupler (OC), which feeds the Stokes and pump beam branches, respectively. A system trigger signal is generated by using a photodiode (PD) that receives a leaked beam from the master laser.

For the Stokes beam generation, 50% of the fs pulse generated at 1.0 µm is amplified by using an FCPA scheme. To this end, the fs pulses are first linearly chirped in a single-mode fibre (SMF, 50 m), after which the chirped pulses are pre-amplified by a core-pumped Yb-doped fibre amplifier (YDFA). The pulses are subsequently modulated by a 20 MHz sinusoidal waveform through an in-line fibre-coupled intensity modulator (FIM), which enables an intensity modulation depth of >20 dB. The average power of the chirped pulses is further amplified to >1.0 W by a double-cladding YDFA (DC-YDFA) that is cladding-pumped by a cost-effective MMFPLD. The amplified laser beam is launched into free space through a fibre collimator (FC1) for pulse compression. The pulse compressor, the black dotted rectangle in Fig. 1a, consists of a grating (G) pair^50^ and an optical telescope (L1 and L2). A slit is placed at the focal plane of L1 to perform narrowband spectral filtering.

The remaining power of the master fs laser is injected into the CWG oscillator, highlighted by the purple dotted rectangle in Fig. 1a, to generate stable self-synchronized laser pulses at 1.5 µm that are ultimately utilized as the pump beam for CRS imaging. The CWG oscillator constructed with an erbium-doped fibre (Er) has a similar structure to the master laser cavity, except that an additional wavelength-division multiplexing (WDM) coupler is used to receive the external injection. Without the external injection, the CWG oscillator operates in the quasi-continuous wave (CW) regime. Once the 1.0 µm laser beam is injected, the CWG oscillator is forced to generate a stable ps pulse train that is stably self-synchronized with the Stokes beam when the cavity length mismatch is less than 200 µm, which results from the combined effect of the NPR pulse compression, XPM and chromatic dispersion (top middle inset of Fig. 1a). Briefly, the injected signal pulse initiates a pulsed perturbation on the quasi-CW laser beam at 1.5 µm through the XPM. This perturbation self-evolves into a stable pulse through NPR compression and circulates in the cavity. The subsequent optical collision between the circulating 1.5 µm pulses and the continuously injected 1.0 µm pulses induces a timing-dependent frequency shift on the 1.5 µm pulse train also through the XPM effect^51–53^. If the 1.5 µm pulse train is slower than the 1.0 µm pulse train, the centre wavelength of the 1.5 µm pulse train is blue-shifted, thus increasing its group velocity in the anomalous dispersion regime. In contrast, a red-shifted centre wavelength will decrease the group velocity of the 1.5 µm pulse train and force the pulse train to synchronize with the 1.0 µm pulse train when it is faster than the 1.0 µm pulses. Figure 1b–e illustrates the temporal evolution of the 1.5 µm pulse train without and with self-synchronization in the cases where the round-trip time (the inverse of the FRR) of the 1.5 µm pulse is slower (Fig. 1b, c) or faster (Fig. 1d, e) than that of the 1.0 µm pulse. The timing reference is 0 s, i.e., the time when the 1.0 µm pulses are injected (not shown). Detailed studies of self-synchronization and its versatile performance are covered in Supplementary Material 3 and 4, respectively. The synchronized laser pulse train at 1.5 µm is then amplified to approximately 1 W by a double-cladding erbium/ytterbium-doped fibre amplifier (DC-EDFA). Here, the FCPA is not utilized, since ps pulses are less sensitive to the fibre nonlinearities than the fs pulses in the Stokes beam. To match the optimal wavelength window of commonly used high-quality microscope objective lenses, i.e., the Olympus UPLSAPO 60XW objective in this case, and the highest sensitivity of the Si photodiode, i.e., typically <1.0 µm, the optical wavelength of the amplified 1.5 µm pulse train is frequency-doubled to the visible regime by second-harmonic generation (SHG) in a periodically poled lithium niobate (PPLN) crystal.

**Fig. 2:**
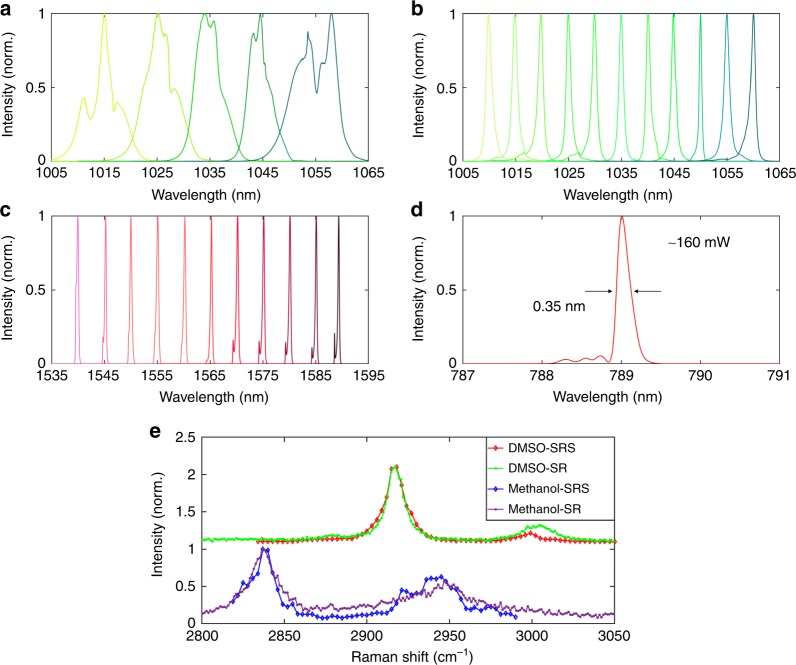
Spectral characteristics of the two-colour pulsed fibre laser. **a** Coarse tuning range of the passively mode-locked fibre laser at 1.0 µm, tuned by the intracavity filter (F1, ~8.0 nm passband). **b** Fine tuning range of the Stokes beam, 1010–1060 nm, tuned by the slit in the pulse compressor, i.e., the black dotted rectangle in Fig. 1a, which has an effective passband of ~1.0 nm. **c** Tuning range of the coherent wavelength generator at 1.5 µm, tuned by the intracavity filter (F2, <1.0 nm passband). **d** Typical SHG spectrum centred at 789 nm, i.e., the pump beam. It is noted that the intensities in all figures have been normalized. **e** Comparison of the SRS spectra and spontaneous Raman (SR) spectra of dimethyl sulfoxide (DMSO) and methanol samples. Note that the spectra of DMSO have been vertically offset for better visualization

Figure 2 shows the spectral performance of the two-colour pulsed fibre laser. The centre wavelength of the master fs laser can be coarsely tuned between 1010 and 1060 nm (Fig. 2a), with a bandwidth of ~8.0 nm defined by the intracavity filter (F1). Fine spectral tuning, i.e., Fig. 2b, is obtained by translating the slit in the pulse compressor, which enables a continuous wavelength scan with an effective bandwidth of ~1.0 nm. On the other hand, the centre wavelength of the CWG oscillator can be continuously fine-tuned from 1540 to 1590 nm with a bandwidth of <1.0 nm (Fig. 2c). Figure 2d showcases a typical SHG spectrum that is frequency-doubled from 1578 nm and has a spectral width of 0.35 nm, corresponding to a transform-limited Gaussian pulse width of 2.6 ps. The average power of the pump beam after the SHG crystal is >160 mW, which is sufficient for CRS imaging^32,35^. It should be noted that an SHG output power of >1 W can potentially be obtained by further optimizing the SHG efficiency of the PPLN crystal^54^. The current two-colour pulsed fibre laser can cover a broad range of Raman resonances in the high-wavenumber region from 2700 to 3550 cm^−1^, including the CH_2_ stretching resonance at 2845 cm^−1^ predominantly associated with lipids and the resonance of cellular proteins at 2920 cm^−1^. To demonstrate the hyperspectral CRS capability, the SRS spectra of standard samples, such as dimethyl sulfoxide (DMSO) and methanol, are measured using this fibre laser source and compared to spontaneous Raman spectra (Fig. 2e).

**Fig. 3:**
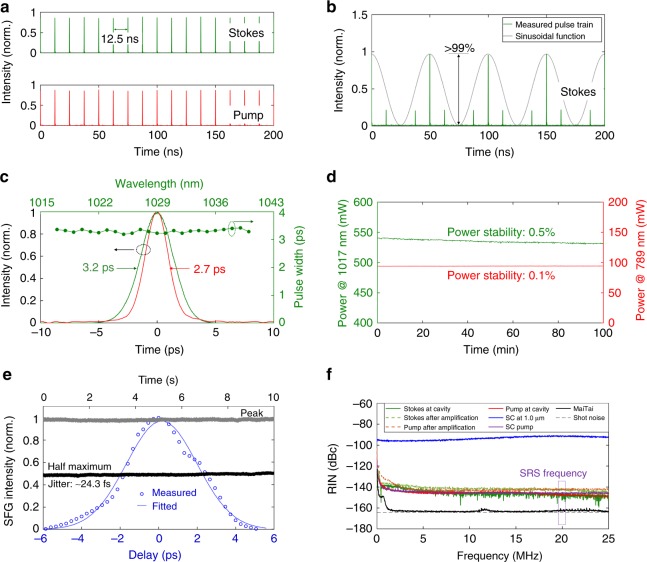
Temporal characteristics of the two-colour pulsed fibre laser. **a** Real-time pulse trains of the Stokes and pump beams at 1017 and 789 nm, respectively, recorded by a 20 GHz real-time oscilloscope. Extended pulse trains (up to 500 µs) of the Stokes and pump beams are also provided in Fig. S6, which details the exceptionally low-intensity noise of this two-colour pulsed fibre laser. **b** Real-time pulse train of the Stokes beam modulated by a 20 MHz sinusoidal function. The modulation depth is higher than 99%. **c** Autocorrelation traces of the pump and Stokes beams and the pulse width stability of the Stokes beam over the tuning range. **d** Long-term power stability over 100 min of both the pump and Stokes beams. In this measurement, the output powers of the pump (789 nm) and Stokes (1017 nm) beams are set to ~100 and ~550 mW, respectively. The root-mean-square (RMS) power fluctuations are only 0.1% and 0.5%, respectively. **e** Optical cross-correlation measurement through sum-frequency generation (SFG). The grey and black curves show the monitored SFG intensities at the peak and half maximum of the optical cross-correlation trace (blue). **f** Comparison of the relative intensity noise (RIN) spectra of the two-colour pulsed fibre laser, a standard solid-state fs laser (Spectra-Physics MaiTai) and a typical supercontinuum (SC) fibre laser

Figure 3a depicts the real-time pulse trains of the pump and Stokes beams, as measured by a 20 GHz real-time oscilloscope, while extended pulse trains up to 500 µs are also shown in Fig. S6. The uniform intensities over the long real-time pulse trains indicate a low-intensity fluctuation, which is crucial for high-quality SRS imaging^32^. The benefit of fibre-based intensity modulation for SRS imaging is presented in Fig. 3b, which shows a modulation depth of more than 99%. This result is also confirmed by a radio-frequency (RF) spectrum measurement (Fig. S7), exhibiting a signal-to-noise (SNR) ratio of 67 dB at 20 MHz. This all-fibre modulation scheme with a large modulation depth is well suited for lock-in detection of SRS signals and is a powerful alternative to cost-intensive free-space acousto- or electro-optic modulation (AOM/EOM) schemes. The pulse widths of both the pump and Stokes beams are measured to be ~2.7 and 3.2 ps, respectively, as shown in Fig. 3c, providing a reasonable compromise between signal strength and molecular sensitivity. Notably, unlike standard OPO-based two-colour lasers that suffer from a large variation in the pulse width when their wavelengths are tuned across a wide range in hyperspectral CRS experiments, our two-colour fibre laser exhibits a constant pulse width over the tuning range, i.e., 3.2 ± 0.06 ps, corresponding to a variation of only 1.8%, which is crucial for high-fidelity spectral data.

Since the modulation transfer from the Stokes beam to the pump beam in SRS is very weak, the noise performance of laser sources is a critical parameter; thus far, balanced detection schemes have been required when fibre-based laser sources are utilized^32,35^. The noise performance of our two-colour pulsed fibre laser is studied on both short- and long-term time scales. To evaluate the power stability over a long time period, which is usually important for long-term imaging of biological samples, the average powers of the pump and Stokes beams are set to approximately 100 and 550 mW, respectively, and then monitored over 100 min (Fig. 3d). Within such a long time period, the fluctuations in the optical power are only 0.1% and 0.5% for the pump and Stokes beams, respectively. The timing jitter between the pump and Stokes pulses is estimated from the intensity fluctuation of a sum-frequency generation (SFG) signal obtained by focusing the beams into a beta barium borate (BBO) crystal^55^. Figure 3e shows an optical cross-correlation trace measured by precisely scanning the delay between the pump and Stokes beams, while the grey and black curves are the SFG intensities monitored over 10 s at two different delays, i.e., 0 ps (peak) and 2 ps (half maximum), respectively. The timing jitter is then calculated from the intensity fluctuation of the SFG signal and the slope of the cross-correlation trace to be approximately 24.3 fs, i.e., ~0.8% of the pulse width. The relative intensity noise (RIN) of the two-colour beams is also characterized and compared with that of a standard solid-state fs laser (Spectra-Physics MaiTai) and a typical SC-based fibre laser (Fig. 3f). At the modulation frequency for SRS imaging, i.e., 20 MHz, the pump and Stokes beams of our fibre laser have similar noise levels, i.e., approximately −147 and −148 dBc/Hz, respectively. Note that this performance characteristic distinguishes our laser source from other designs, where one of the two-colour beams usually exhibits degraded noise performance after the nonlinear conversion processes^56,57^; see Supplementary Material 6. Here, these two-colour beams exhibit equally low RIN levels, since the RIN of the pump beam has been improved by 50 dB compared with previous implementations (Table 1)—a key requirement for high-quality SRS imaging without the need for balanced detection^32,35^. It is noted that the intensity noise level of our laser system is still worse than that of standard solid-state lasers (Fig. 3f), which can be attributed to pump laser noise, amplified spontaneous emission (ASE) noise and environmental fluctuations. The intensity noise level, however, could be further improved by using electronic feedback schemes^58^. Currently, the entire fibre laser system is loosely placed on an optical table over an area of ~80 × 85 cm^2^, where no particular efforts have been made to minimize its size.

**Table 1:** Performance of all-fibre lasers enabling SRS imaging

Fibre laser sources	Current work	ASFL^18^	SSFSFL^29^	SCFL^32^
Performances				
Synchronization scheme	Passive	Active	Passive	Passive
Power [pump, Stokes], mW	[160, 1000]	[12, 15]	[120, 10]	[75, 120]
Long-term power stability, %	<0.5			
RIN [pump, Stokes], dBc/Hz	[−147, −148]			~[−140, −90]^a^
Pulse width [pump, Stokes], ps	[2.7, 3.2]	[4, 3]	~[0.7, 0.47 ± 0.11]	[4.0, 1.0]
Pulse width variation, %	<1.8%		~25	
Wavenumber region, cm^−1^	2700–3550	2600–3411	2330–3330	2670–3630
Timing jitter, fs	24.3	227		<24
Intensity modulation depth, %	>99			
SRS imaging speed, µs/pixel	6.4	3000^b^	3000^b^	<5^b^

^a^The RIN of the Stokes beam was not provided in the reference; here, it was estimated from the results of duplicated experiments (Fig. 3f). Note that a better value could have been obtained in the reference

^b^The SRS imaging was performed with additional balanced detection to suppress the intensity noise

**Fig. 4:**
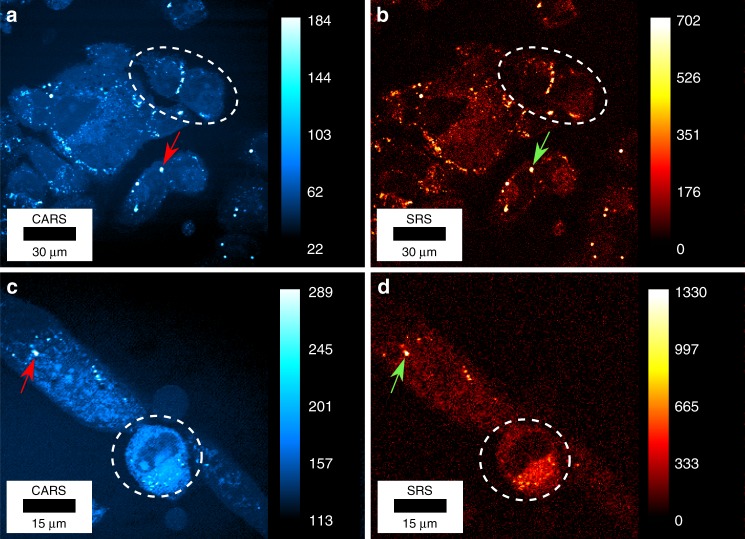
CARS and SRS images of living human cells. **a**, **b** CARS and SRS images of living osteosarcoma (U2OS) cells acquired simultaneously. The focal powers are set to 33 and 74 mW for the pump and Stokes beams, respectively. The pixel dwell time is 12.5 µs, and the time constant for the lock-in amplifier is 2 µs. **c**, **d** CARS and SRS images of a living differentiating primary myoblast (PMD). Here, a 2× zoom-in is applied to reveal more details of the cells. The focal powers are 25 and 47 mW for the pump and Stokes beams, respectively, the pixel dwell time is 51.2 µs, and the lock-in time constant is 20 µs. The arrows indicate the position of selected lipid droplets inside the cells

After spatially and temporally overlapping the pump and Stokes beams using a dichroic mirror (DM) and an optical delay line (DL), the combined beams are coupled into our custom-built laser scanning microscope (see “Methods” and Fig. S1). To demonstrate the unique capabilities of our two-colour pulsed fibre laser, we simultaneously acquired CARS and SRS images of biological samples ranging from single cells to tissue sections. Figure 4a–d shows CARS and SRS images of living human osteosarcoma (U2OS) cells (Fig. 4a, b) and living differentiating primary myoblast (PMD) cells (Fig. 4c, d). Note that no averaging has been applied to any of the images in the current manuscript. Here, the CH_2_ stretching mode at 2845 cm^−1^ is utilized as the chemical contrast to mainly highlight lipid-rich regions within the cells such as lipid droplets, which can be observed as areas of high signal intensity (i.e., bright spots indicated by select arrows). For the U2OS sample, the focal powers of the pump and Stokes beams were set to 33 and 74 mW, respectively, while maintaining a pixel dwell time of 12.5 µs (see Table S5) and a time constant of 2 µs for the lock-in amplifier (LIA). Again, it should be noted that these images were acquired without balanced detection for noise cancellation, demonstrating that our two-colour pulsed fibre laser facilitates high-contrast and fast live-cell imaging with a pixel dwell time of a few µs. Additional z-scans of the U2OS cells are shown (both CARS and SRS) in Supplementary Videos S1 and S2. Next, we imaged living PMD cells (Fig. 4c, d), which have lower lipid content than U2OS cells. Even though the focal power was reduced to 25 and 47 mW for the pump and Stokes beams, respectively, lipid droplets (indicated by the arrows) and the plasma membrane highlighted by the 2845 cm^−1^ resonance can easily be identified (Fig. 4c, d). The contribution of the non-resonant signal results in a higher overall signal in the CARS images than in the SRS images. On the other hand, the SRS images with SNRs of 45.7 and 16.7 in Fig. 4b, d, respectively, specifically highlight the chemical compartments, as indicated by the white dotted circles, that are resonantly responding to the corresponding Raman resonance, which is predominantly associated with aliphatic CH_2_ groups in lipids.

**Fig. 5:**
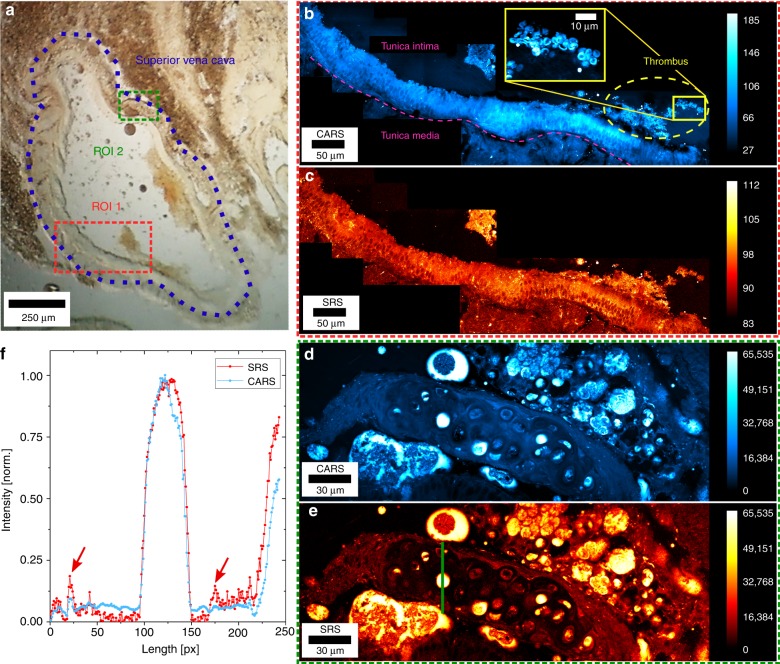
CARS and SRS images of a mouse superior vena cava tissue cryo-section. **a** Bright-field image of the mouse superior vena cava tissue cryo-section. The green and red regions of interest (ROIs) indicate the positions where CRS imaging is applied, respectively, and the violet dotted line displays the general location of the superior vena cava. **b**, **c** CARS and SRS data acquired from the red outlined area, respectively. Here, we can discriminate between tunica media and tunica intima with excellent contrast and visualize a thrombus containing red blood cells (see the yellow inset of (**b**)), which adhere to the arterial wall. A pink dotted line is inserted in (**b**) to depict the elastica interna, separating both areas from each other. **d**, **e** CARS and SRS images of the green outlined area detailing the lipid-rich tunica media of the superior vena cava. The optical powers are set to 33 and 74 mW for the pump and Stokes beams, respectively. The pixel dwell time is 6.4 µs, and the lock-in time constant is 2 µs. The modulation frequency of the Stokes beam is 20 MHz. **f** Corresponding line scans of the images (**d**) and (**e**). The location is indicated by the green line in image (**e**). Here, we compare the CARS and SRS signals in a lipid-rich area of the tunica media. Small lipid regions are revealed by SRS due to its enhanced chemical sensitivity (red arrows). The signal is normalized to the highest signal intensity of both channels

We also imaged tissue sections of a 2-month-old mouse with severe combined immunodeficiency. Figure 5 shows the CARS images and the corresponding SRS images of a cross-section of superior vena cava tissue prepared on a thin glass cover slip using a cryo microtome. Figure 5a displays a bright-field image of the mouse tissue section, captured at ×4 magnification. Here, the superior vena cava can be identified in the middle of the image. Two regions of interest (ROIs) were chosen for CRS imaging, as depicted by the red (ROI 1) and green (ROI 2) dashed rectangles, where multiple CRS images were taken. The sequentially acquired images were stitched together to extend the field of view (FOV) and provide a wider context of the sample’s morphology. In Fig. 5b, c, such an extended FOV can be seen in ROI 1. Here, we observe the change from tunica media to tunica intima and a thrombus containing red blood cells that are attached to the vascular wall. The yellow inset provides an enlarged view of the area of the red blood cells. Figure 5d, e shows a lipid-rich area of the tunica media in the same acquisition mode. The line profile (green) across the tunica media **(**Fig. 5f) shows the intensity profiles of the CARS and SRS images and is normalized to the maximum intensity present in the line scan. Indicated by the two red arrows, we observe regions in which the non-resonant background masks small features in the CARS image that can be revealed in the SRS image due to the enhanced chemical sensitivity. In Supplementary Videos S3 and S4, we utilize the z stage to move the focus through the sample with a step size of 4 µm for CARS and SRS imaging, respectively.

**Fig. 6:**
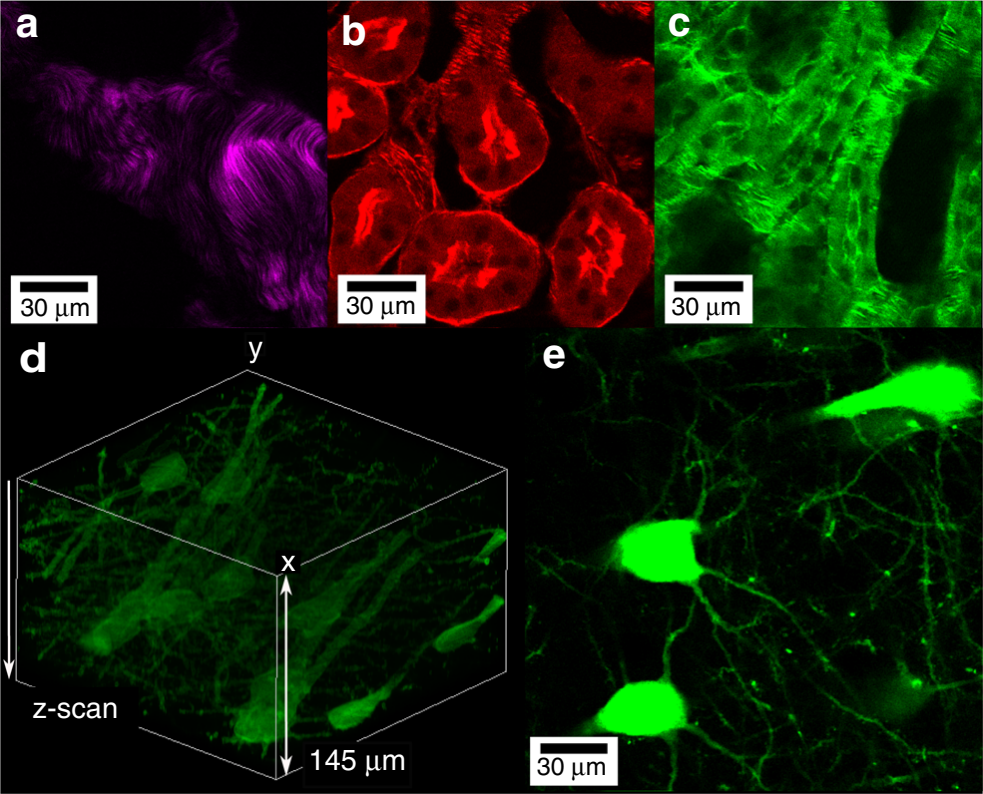
SHG imaging of a mouse tail sample and two-photon fluorescence imaging of kidney and brain tissue sections. **a** SHG image of a non-stained mouse tail slide. The applied polarization is controlled by a half-wave plate and a quarter-wave plate. Focal power of ~20 mW is applied to the sample utilizing the FCPA output of the Stokes beam (bandwidth of ~8 nm, pulse width of ~200 fs). **b**, **c** Two-photon excited fluorescence images of a mouse kidney section (Thermo Fisher Scientific FluoCells® prepared slide #3) using ~20 mW of focal power. **d**, **e** Two-photon excited fluorescence images of a 200-µm-thick mouse brain tissue sample prepared between two glass slides, where the mouse expresses yellow fluorescent protein (YFP) in layer-V pyramidal neurons (Thy1-YFP H-line)^59^. **d** A 3D-volume composition of the data acquired via z-scanning over 145 µm (montage created with the Fiji volume viewer), while **e** is a single frame illustrating the neuron distribution. Here, focal power of ~50 mW is applied

The versatility of our laser source is also demonstrated by the fact that the Stokes beam after the FCPA stage can be used as a fs laser by removing the filtering slit to serve as a source for SHG and two-photon excited fluorescence imaging. In this case, the central wavelength is set to 1025 nm with an 8 nm bandwidth, corresponding to a pulse width of ~200 fs. To demonstrate this versatility, an unstained mouse tail tissue section was imaged by SHG with different polarization states of the laser; one particular polarization is shown in Fig. 6a. The SHG signal is mostly generated by collagen in the tendon tissue, while the signal from the surrounding muscle tissue is relatively weak. We then imaged fluorescently stained samples, such as a tissue section of a mouse kidney and the neurons of a mouse brain section (Fig. 6b–e). In Fig. 6b, the fluorescence emission of Alexa Fluor 568 phalloidin shows the filamentous actin cytoskeleton prevalent in the glomeruli and brush border, while in Fig. 6c, the fluorescence signal of Alexa Fluor 488 (staining wheat germ agglutinin) indicates glomeruli and convoluted tubules. These two images were separated with two bandpass filters centred at 519 and 600 nm, respectively. Figure 6d, e is two-photon excited fluorescence images of a 200-µm-thick mouse brain tissue sample prepared between two glass slides, where the mouse expresses yellow fluorescent protein (YFP) in layer-V pyramidal neurons (Thy1-YFP H-line)^59^. Here, the same objective lens (Olympus UPLSAPO 60XW) was utilized to perform a z-scan of the sample over a depth range of 145 µm using a step size of 1.0 µm (Fig. 6d). Figure 6e shows a single frame illustrating the neuron distribution. It should be pointed out that the imaging depth is limited to the sample thickness and working distance of the objective lens (~280 µm). Hence, the laser source has multimodal imaging capabilities, especially because the fundamental wavelength (1570 nm) used as the pump beam for CRS imaging in this work is a potential source for three-photon excited fluorescence, which is not shown in this work.

## Discussion

We have demonstrated a ps two-colour fibre laser that operates with low noise, sufficient power, and wide wavelength tunability in the high-wavenumber region from 2700 to 3550 cm^−1^ commonly used for CRS microscopy. Due to CWG utilizing XPM, both the pump and Stokes beams exhibit very low relative intensity fluctuations of less than −140 dBc/Hz, which enables us to use this compact and robust laser source for high-speed SRS imaging without balanced detection. This improvement is significant for successfully translating CRS and related nonlinear optical imaging technologies to clinical applications, while prior implementations have required either a long signal integration time or balanced detection to eliminate the exceeding laser noise. As a result, we obtain high-contrast, fast-scanning CRS images of living human cells and mouse superior vena cava tissue. Furthermore, with the additional benefit of the fs chirped-pulse amplification in the Stokes beam branch, we are able to utilize multiple optical contrast mechanisms simultaneously by conducting multimodal nonlinear optical imaging, including CARS, SRS, SHG and two-photon fluorescence excitation. It is anticipated that the remarkably low noise of this two-colour pulsed fibre laser system over both short- and long-term time scales will be essential for achieving high-quality images of living samples with low pixel dwell times, e.g., living cells and organisms. This scheme can be extended to other wavelength windows through the same CWG method, e.g., 900 nm (neodymium-doped fibre), 1100–1500 nm (bismuth-doped fibre) and 1700–2000 nm (thulium-doped fibre). Fibre-based laser beam delivery is also compatible with many other optical imaging systems; e.g., it can easily be attached to any confocal microscope with suitable detectors and integrated with existing endoscopy systems^60,61^ through fibre connections to realize compact and user-friendly CRS imaging for use in surgery and diagnostics^62^.

The current implementation can potentially be upgraded in the following two ways: (1) for fast spectroscopic imaging, the wavenumber tuning speed can be largely increased by replacing the existing spectral filters with electronically controlled all-fibre Fabry-Pérot filters that can easily operate at speeds of >100 kHz and have widely been used to build fast wavelength-swept laser sources for high-speed optical coherence tomography^63^; (2) to fully utilize the appealing advantages of fibre lasers for flexible beam delivery, the frequency doubling of the pump beam, which is currently performed in free space, can be replaced with a fibre-integrated PPLN module^64^, resulting in an even more compact and robust system.

## Methods

### Customized laser scanning microscope

Two xy-scanning galvanometric mirrors (Cambridge Technology 6215H) raster scanned the laser focus across the sample. A 60× water immersion objective lens (Olympus UPLSAPO 60XW, NA = 1.2) was used to focus the beams into the sample, while its back focal plane was filled by a scanning telescope. For a larger FOV and the capability to acquire 3D images, the sample was mounted on a motorized xyz-stage. The CRS signals were collected in the forward direction through a condenser lens (Olympus U-AAC, NA = 1.4). The CARS signal was isolated from the excitation laser wavelengths by a DM (Semrock LP02-785RU) and a filter set (Semrock BSP01-785R x2, FF01-655/40-25) and finally detected by a photomultiplier tube (Hamamatsu Photonics H9656-20). The pump beam was separated by the same DM and a filter (Semrock FF01-855/210-25, FF01-950/SP) and focused onto a Si photodiode (APE Berlin), which was fed into a fast lock-in amplifier (LIA, customized from APE Berlin) in combination with the reference modulation frequency delivered by a function generator. All of the resulting electronic signals were acquired by a data acquisition card (National Instruments PCI-6110S) and analyzed by the MATLAB program ScanImage (Version 3.8.1)^65^. The fast intensity modulation of the Stokes beam was achieved in the following way: a system trigger signal was generated from a leaked beam by using an InGaAs-photodiode (Thorlabs DET01-CFC). This 80 MHz pulse signal was then passed through an amplifier and a frequency divider, which divides the frequency by a factor of 4. This signal then triggered a function generator (HP 8116A) that generates a 20 MHz sinusoidal frequency, which was finally fed to the fast fibre-based intensity modulator (iXblue NIR-MX-LN-10) in the Stokes beam path. The synchronized trigger output of the function generator was used as the reference signal for the fast LIA.

### Image processing

The images were processed with the open-source program Fiji. Here, we used the standard look-up table “cyan hot” for the CARS images, “red hot” for the SRS images, and “red”, “green” and “magenta” for two-photon and SHG imaging. The black and white values were arbitrarily chosen for optimal contrast. The enlarged overview images of the mouse tissue were constructed from the raw data utilizing the plugin “Stitching” in Fiji.

### Sample preparation

(1) Cultivation of the cells: human bone osteosarcoma epithelial cells (U2OS) were cultured in Dulbecco’s modified Eagle’s medium (DMEM, Thermo Fisher) containing 10% foetal bovine serum (Thermo Fisher) and penicilium-streptomycin (P/S, Sigma Aldrich) on a standard petri dish. Differentiating primary myoblast cells were cultivated in DMEM containing 5% horse serum (Thermo Fisher) and P/S on petri dishes coated with an extracellular matrix (Sigma Aldrich) at 1:500. All cells were cultured in a humidified incubator at 37 °C, 5% O_2_ and 5% CO_2_. Before imaging, the cell medium was replaced by phosphate-buffered saline (PBS, Sigma Aldrich). (2) Tissue samples: the mouse superior vena cava tissue cryo-section was extracted from a 2-month-old immunodeficient mouse. The tissue was embedded in paraffin and cut into 50-µm-thick sections with a cryo microtome; the mouse tail section was harvested from a C57BL/6 mouse ~3-month old, housed and maintained in the Animal and Plant Care Facility, The Hong Kong University of Science and Technology (HKUST). All animal experiments were approved by the HKUST Animal Ethics Committee. The mouse kidney section was ordered from Thermo Fisher Scientific (FluoCells® prepared slide #3). One-month-old Thy1-YFP H line mice were perfused with 4% paraformaldehyde. Brain tissue was removed and post-fixed overnight. The brain tissue was further cut into 200-µm-thick sections by a vibratome for two-photon imaging. Each cut was collected on a glass coverslip (No 1.5, Carl Roth) and stored in PBS. All of the experiments using these samples were approved and performed in accordance with The University of Hong Kong Committee on the Use of Live Animals in Teaching and Research guidelines.

## Supplementary information

Supplementary Material
