# Supplementary material for: High-contrast, fast chemical imaging by coherent Raman scattering using a self-synchronized two-colour fibre laser
Source: Light Sci Appl. 2020 Feb 24;9:25. doi: 10.1038/s41377-020-0259-2 (PMC7039946; doi:10.1038/s41377-020-0259-2)
Supplement: Supplementary file 1 — Supplementary Material [file 41377_2020_259_MOESM1_ESM.docx]

**Supplementary Materials for:**

**High-contrast, fast chemical imaging by coherent Raman scattering using a self-synchronized two-colour fibre laser**

Cihang Kong^1,2,†^, Christian Pilger^2,†^, Henning Hachmeister^2,†^, Xiaoming Wei^1,3,*^, Tom H. Cheung^4^, Cora S. W. Lai^5^, Nikki P. Lee^6^, Kevin. K. Tsia^1^, Kenneth K. Y. Wong^1,*^ and Thomas Huser^2,*^

^1^Department of Electrical and Electronic Engineering, The University of Hong Kong, Pokfulam Road, Hong Kong, China

^2^Biomolecular Photonics, Department of Physics, University of Bielefeld, Universitätsstr, 25, 33615 Bielefeld, Germany

^3^Currently at Division of Engineering and Applied Science, California Institute of Technology, 1200 East California Boulevard, Pasadena, California 91125, USA

^4^Division of Life Science, The Hong Kong University of Science and Technology, Hong Kong, China

^5^Department of Physiology, Li Ka Shing Faculty of Medicine, The University of Hong Kong, Pokfulam, Hong Kong, China

^6^Department of Surgery, The University of Hong Kong, Pokfulam Road, Hong Kong, China

^†^These authors contributed equally to the paper

^*^Correspondence should be addressed to X.W. ([xmwei@caltech.edu](mailto:xmwei@caltech.edu)), K.K.Y.W. ([kywong@eee.hku.hk](mailto:kywong@eee.hku.hk)) or T.H. ([thomas.huser@physik.uni-bielefeld.de](mailto:thomas.huser@physik.uni-bielefeld.de))

1. **Typical schemes of fibre lasers for CRS imaging**

Here, we briefly summarize the schemes of fibre lasers that have recently been proposed and demonstrated for CRS imaging, while their performances are analysed in **Table S1**, where the areas marked in grey colour indicate their current drawbacks.

- - **Hybrid fibre laser (HFL)** usually combines a standard solid-state laser (typically Ti:sapphire laser) with a fibre laser for the pump and Stokes beams of CRS imaging, respectively^1-4^. They are synchronized through active feedback methods, e.g., two-photon detection associated with intracavity electro-optic modulation (EOM)^1,3^ and time-lens^2,4^. Inheriting from the low noise performance of solid-state lasers, HFLs work at a reasonably low level of noise. As a part of HFLs, however, solid-state lasers are expensive, and have large footprints. More importantly, the maintenance needs professional skills.
  - **Actively synchronized fibre laser (ASFL)** utilizes the electronic modulation and control to synchronize two fibre lasers, e.g., the phase-locked loop (PLL)^5^ and EOM^6^. Although it is relatively easy to implement, this kind of fibre lasers has exceeding noise, and it thus requires a very long pixel dwell time for SRS imaging, typically >1 ms^5,6^ even with balanced detection.
  - **Parametric wavelength conversion fibre laser (PWCFL)** is typically implemented by pumping a long piece of highly-nonlinear fibre (HNF) with a pulsed fibre laser, and certain new frequencies are generated through the degenerate four-wave-mixing (FWM) under phase matching condition^7-14^. The configuration of PWCFLs can be either a parametric amplifier or oscillator, and both are usually compact. However, they easily operate at a high level of noise generated from either the pump itself or the seed, i.e., less than optimal for high quality SRS imaging^9^.
  - **Soliton self-frequency shift fibre laser (SSFSFL)** relies on the Raman soliton generation in a short piece of HNF by using fs laser pulses^15-20^. It typically needs a dedicated dispersion management for compressing the pumping pulse width to about 100 fs. Due to a low power efficiency, the optical power of the red-shifted component, usually served as the Stokes beam of CRS imaging, is only a few mW. In addition, the Raman soliton usually has a broad spectrum and thus is not ideal for a high spectral resolution of CRS imaging.
  - **Supercontinuum (SC) fibre laser (SCFL)**, similar to the PWCFL and SSFSFL, usually pumps a piece of HNF with a pulsed fibre laser^21-28^. On the other hand, it is different from the PWCFL and SSFSFL that SC generation lacks the spectral selection, and it simply broadens the optical spectra of pumping pulses into super broad ones. SCFLs usually have a low power spectral density, and external amplifiers are usually required to boost the optical power at the targeted wavelengths^23,26^. SCFLs can also have a high level of noise, particularly when it operates in the long pulse regime where FWM and Raman scattering play key roles in the spectral broadening^29,30^. To perform fast SRS imaging, the balanced detection has recently been proposed to cancel the exceeding noise of SCFLs^23,26^.

**Table S1 Fibre lasers for CRS imaging**

|  | **HFL** | **ASFL** | **PWCFL** | **SSFSFL** | **SCFL** | **Current work** |
| --- | --- | --- | --- | --- | --- | --- |
| **Pulse width** | <1 ps^1^  1-10 ps^2-4^ | <4 ps^5^  CW^6^ | 1–10 ps^7,9,13^  10–100 ps^8,10-12,14^ | <1 ps^15-20^ | <1 ps^25^  1–10 ps^21-24,26-28^ | ~3 ps |
| **Pump beam power, mW** | >1000^1,3^ | 12^5^  ~500^6^ | <100^7,8,11,12^  100–200^9,10,13,14^ | 1–10^19^  10–100^15-17,20^ | <10^24^  10–200^21-23,26-28^  >200^25^ | >160 |
| **Stokes beam power, mW** | 100–500^1-4^ | ~3^6^, 15^5^ | <100^10^  100–200^14^  >200^7-9,12,13^ | <1^19^  1–10^15,16,20^  >10^17^ | <10^21^  10–200^22-28^  >200^24,25^ | >1000 |
| **Spectral range, cm^-1^** | 1600–2850^2,4^  2700–3100^1,3^ | 2600–3411^5^  750–3150^6^ | 1000–2000^7^  2700–3100^9-11,13,14^  1000–5000^8,12^ | 0–2500^15^  2300–3300^16,20^  700–4000^17,19^ | 1000–2000^24^  2600–3700^23,26,27^  1000–5000^21,22,25,28^ | 2700–3550 |
| **Noise level*** | Low | High | High | High | High | Low |
| **SRS imaging or not?** | Yes^1,2,4^ | Yes^5,6^ | No | Yes^19,20^ | Ye^23,26-28^ | Yes |
| **SRS imaging speed, µs/pixel** | 2^4^, >200^1^ | 3000^5^, >5000^6^ | NA | 3000^20^, 20000^18^, 30000^19^ | <5^23,26^ | >6.4 |
| **Balanced detection or not?** | No | Yes^5,6^ | NA | Yes^20^ | Yes^23,26^ | No |
| **Use of solid-state laser** | Yes | No | No | No | No | No |
| **Biological samples for SRS imaging** | Tobacco BY-2 cell, cultured HeLa cell^1^, mouse brain, mouse skin^2^, PC3 cells^4^ | Geranium phaeum stem^6^, HeLa cell^5^ | NA | Aquatic plant^20^, mouse brain, blood cells^19^, polystyrene bead^18^ | Mouse skin^23^, human surgical specimens^26^ | Living osteosarcoma cell, living differentiating primary myoblast cell, mouse superior vena cava tissue, mouse brain |
| ***Referring to the noise level of solid-state lasers that are typically shot noise limited** | | | | | |  |

1. **Full configuration of the self-synchronized two-colour pulsed fibre laser and CRS microscope**

**Figure S1** illustrates the full schematic diagram of the self-synchronized two-colour pulsed fibre laser and custom-built laser scanning microscope, while **Tables S2** and **S3** list the key components that have been used in this work and the prices of components used in the master laser, respectively. The two-colour pulsed fibre laser starts from a passively mode-locked fs fibre laser at 1.0 µm, i.e., the master laser of the laser system. Fs pulses are generated by the nonlinear polarization rotation (NPR) technique in the all-normal dispersion regime^31^. The mode-locked fibre laser has a very compact design, as shown in the top left corner of **Fig. S1**. The whole fibre cavity is looped by a fibre-based optically integrated module (OIM, Optizone Tech.), which not only couples the pump laser beam from a fibre-coupled pump laser diode (FPLD, II-VI LC96Z400) and partially extracts the mode-locked pulse, but also ensures a polarization-sensitive and unidirectional operation, which is crucial for a self-started mode-locking. The gain medium is a short piece of single-cladding ytterbium-doped fibre (Yb, Thorlabs YB1200-4/125, about 25 cm in length). Throughout this work, the fusion splicing is utilized to connect different fibres together. The splicing loss is typically less than 0.1 dB. For NPR mode-locking, a compact drop-in polarization controller (PC, General Photonics PolaRite, 25x40x75 mm in size) is employed to adjust the state of polarization (SOP) of the laser beam, which works by placing a portion of the bare fibre into its slot. The wavelength tuning is achieved by using a tunable filter (F1, Semrock LL01-1064-12.5), which has a bandwidth of about 4.0 nm and a tuning range of about 50 nm (1010 – 1060 nm). It is sandwiched by two fibre collimators (FC, Thorlabs TC18APC-1064, not labeled in **Fig. 1a** and **Fig. S1**). It is noted that an all-fibre laser cavity can potentially be obtained by using a fibre-coupled tunable filter, e.g., fibre optic electronically tunable filter from Agiltron Inc.. A photodiode (PD1, Thorlabs DET01-CFC) is used to receive a leaked laser beam from F1 and convert it into an electronic signal that serves as the trigger signal of a function generator (FG, HP 8116A). In this way, the FG can generate synchronized electronic signals with a tunable phase shift for the modulation of the Stokes beam and lock-in detection in SRS imaging, i.e., a sinusoidal waveform in this case. The cavity length of the master laser has been optimized for a fundamental repetition rate (FRR) of 80 MHz, i.e., about 2.5 m. A higher FRR can also be obtained by simply cutting the intracavity fibre. The output of the master laser is split into two parts through a 50:50 fibre optic coupler (OC, Optizone Tech.), which are subsequently launched to the pump and Stokes beam branches, respectively.


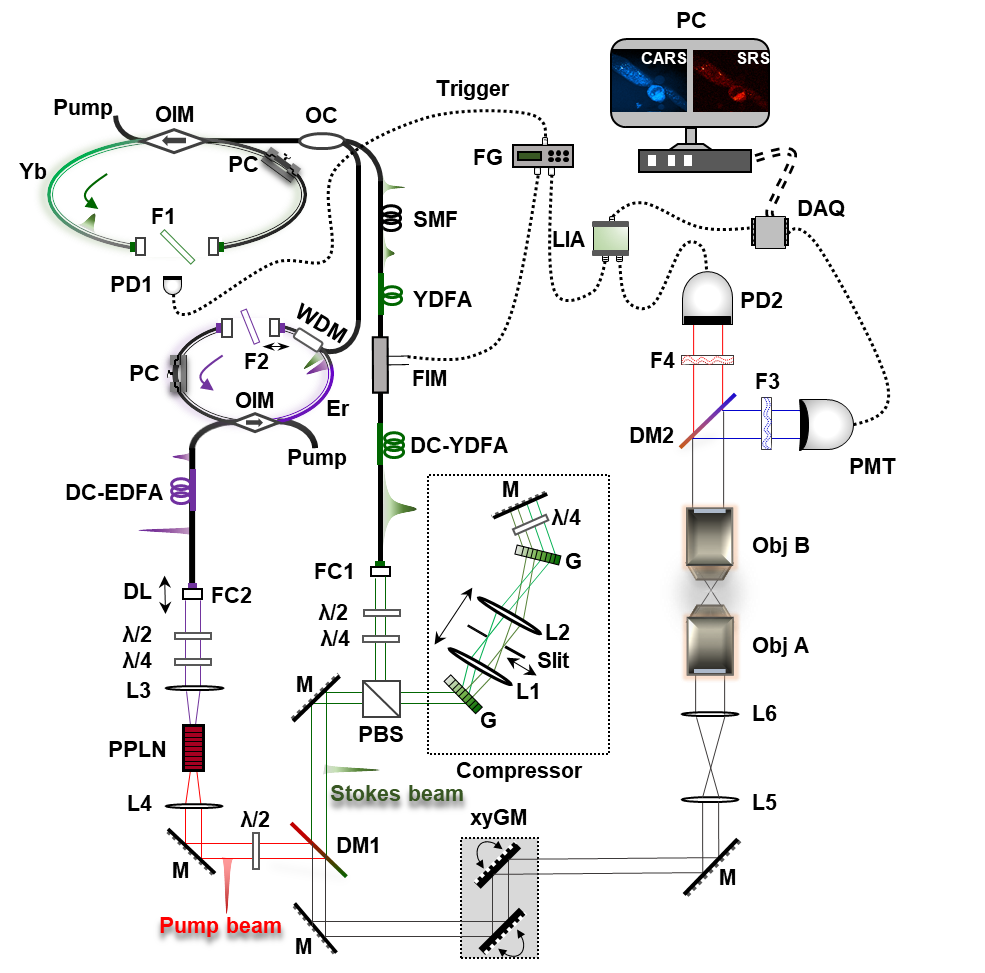


**Figure S1** | **Full schematic diagram of the self-synchronized two-colour pulsed fibre laser and custom-built laser scanning microscope**.

**Stokes beam generation**. The optical power of the Stokes beam is boosted through a fibre chirped-pulse amplification (FCPA) scheme^32^ that composes of **pre-chirping**, **pre-amplification**, **intensity modulation**, **high power amplification** and **pulse compression**. One output port of the OC is directly spliced with a piece of single-mode fibre (SMF, Corning HI 1060, 50 m), which linearly chirps the fs pulse to reduce its peak power. The chirped pulse is subsequently pre-amplified to about 100 mW by a homemade single-cladding ytterbium-doped fibre amplifier (YDFA), which composes of a highly-doped Yb fibre (Thorlabs YB1200-4/125, ~25 cm), a 980/1030 nm wavelength-division multiplexing (WDM, Haphit) coupler, a fibre isolator (Haphit) and a FPLD at 980 nm. A fibre-coupled intensity modulator (FIM, iXblue NIR-MX-LN-10) is used to modulate the Stokes pulse train with a 20 MHz sinusoidal waveform from the FG, which is synchronized with the 80 MHz pulse train. This FIM can provide a large modulation depth of >20 dB, better than free-space schemes, typically 80%^33^. The modulated pulse train is fed into a high power double-cladding YDFA (DC-YDFA) to further increase the average power. The configuration of the DC-YDFA is similar to that of Ref. 34. In brief, a multimode signal/pump combiner (SPC, LightComm) is used to combine the single-mode signal and multimode pump beams, respectively delivered by SMF and multimode fibre (MMF, Nufern MM-S105/125-0.22NA, core/cladding sizes of 105/125 μm). The leading fibre of the SPC output is a double-cladding fibre (DCF, CorActive DCF-UN-10/125-080) that has been optimized for the numerical aperture (NA) of the double-cladding Yb fibre (DC-Yb) with an inner core diameter of 10 µm (iXblue IXF-2CF-Yb-O-10-130-NH-V2.0, 1.5 m). The pump laser of the DC-YDFA is a multimode fibre-coupled pump laser diode (MMFPLD, QPhotonics QSP-975-10) that provides a maximum power of about 10 W at 975 nm. The unabsorbed pump power is extracted from the optical fibre by recoating the rear end of the DC-Yb fibre with the UV-curable optical adhesive that has a higher refractive index of 1.56 (Thorlabs NOA63). After DC-YDFA, the Stokes laser beam with an average power of >1 W is launched into the free space through a fibre collimator (FC1, Thorlabs TC18APC-1064) for pulse compression. As shown in the black dotted rectangle of **Fig. S1**, the pulse compressor is mainly constructed by a grating (G) pair (Lightsmyth LSFSG-1000-3212-94) and an optical telescope (L1 and L2, Thorlabs AC508-075-B). The compression ratio can be changed by translating the optical telescope. Here, a translational slit is placed at the focal plane of L1 as a spectral filter that provides an effective bandwidth of about 1.0 nm for wavelength tuning, see **Fig. 2b**. It should be emphasized that a fibre-based pulse compressor can potentially be implemented by using chirped fibre gratings^35,36^, while a fibre-based spectral filtering can be obtained by using fibre-coupled tunable filters^37^.

**Pump beam generation**. The other 50% output of the OC is injected into a laser cavity for coherent wavelength generation (CWG) through a 1030/1580 nm WDM. The main function of the CWG cavity is to generate self-synchronized stable ps pulses at 1.5 µm, and the principle is detailed in **Section 3**. The CWG cavity is constructed by a fibre-based OIM. A piece of single-cladding erbium-doped fibre (Er, Thorlabs ER80-8/125, 0.5 m) serves as the gain medium, which is core-pumped by an FPLD at 980 nm. The SOP of the laser beam inside the CWG cavity is controlled by another drop-in PC. The oscillating wavelength of the CWG cavity is tuned by using a tunable narrowband filter (F2, BAAR, <1.0 nm bandwidth) that is sandwiched by two FCs (not labeled in **Figs. 1a** and **S1**). The output of the CWG cavity is amplified by a double-cladding erbium/ytterbium-doped fibre amplifier (DC-EDFA), which has a similar schematic as that of the DC-YDFA described before. The gain medium is a piece of heavily-doped double-cladding erbium/ytterbium-doped fibre (DC-EY, CorActive DCF-EY-10/128) that is pumped by another high power MMFPLD at 975 nm. The output of the DC-EDFA is launched into the free space through a fibre collimator (FC2), which is mounted on a linear translation stage as an optical delay line (DL) for the temporal overlapping of the pump and Stokes beams. An achromatic lens (L3, Thorlabs AC508-075-C, 75 mm focal length) is utilized to focus the high power laser pulse into a periodically poled lithium niobite (PPLN, Covesion MSHG1550-0.5) crystal for frequency doubling, e.g., from about 1580 nm to 790 nm (**Fig. 2d**). After the PPLN, another achromatic lens (L4, Thorlabs AC254-125-B) is employed to collimate the laser beam with a matched beam size, which is subsequently combined with the Stokes beam through a dichroic mirror (DM1, Thorlabs DMLP950). The spatially and temporally overlapped laser beams are finally launched to the custom-built laser scanning microscope, which has been described in detail in **Methods**.

**Table S2 Key components**

| Symbol | Description | Model number |
| --- | --- | --- |

| **Fibre components** |
| --- |

| DCF | Double-cladding fibre | CorActive DCF-UN-10/125-080 |
| --- | --- | --- |
| DC-EY | Double-cladding erbium/ytterbium-doped fibre | CorActive DCF-EY-10/128 |
| DC-Yb | Double-cladding ytterbium-doped fibre | iXblue IXF-2CF-Yb-O-10-130-NH-V2.0 |
| DC-EDFA | Homemade double-cladding erbium/ytterbium-doped fibre amplifier | Gain fibre: CorActive DCF-EY-10/128 |
| DC-YDFA | Homemade double-cladding ytterbium-doped fibre amplifier | Gain fibre: iXblue IXF-2CF-Yb-O-10-130-NH-V2.0 |
| EDFA | Homemade single-cladding erbium-doped fibre amplifier | Gain fibre: Thorlabs ER80-8/125 |
| Er | Single-cladding erbium-doped fibre | Thorlabs ER80-8/125 |
| MMF | Multimode fibre | Nufern MM-S105/125-0.22NA |
| MMFPLD | Multimode fibre-coupled pump laser diode | QPhotonics QSP-975-10 |
| OC | 50:50 fibre optic coupler | Optizone Tech. |
| OIM | Optically integrated module | Optizone Tech. |
| PC | Polarization controller | General Photonics PolaRite |
| Pump | Fibre-coupled pump laser diode (FPLD) | II-VI LC96Z400 |
| SMF | Single-mode fibre | Corning HI 1060 |
| SPC | Signal/pump combiner | LightComm |
| WDM | 980/1030 nm, 980/1580 nm, and 1030/1580 nm wavelength-division multiplexing couplers | Haphit |
| Yb | Single-cladding ytterbium-doped fibre | Thorlabs YB1200-4/125 |
| YDFA | Homemade single-cladding ytterbium-doped fibre amplifier | Gain fibre: Thorlabs YB1200-4/125 |

| **Free-space components** |
| --- |

| DM1 | Dichroic mirror | Thorlabs DMLP950 |
| --- | --- | --- |
| DM2 | Dichroic mirror | Semrock LP02-785RU |
| FC1 | Fibre collimator | Thorlabs TC18APC-1064 |
| FC2 | Fibre collimator | Thorlabs F260APC-1550 |
| F1 | Broadband tunable filter | Semrock LL01-1064-12.5 |
| F2 | Narrowband tunable filter | BAAR |
| F3 | Filter set | Semrock BSP01-785R x2, FF01-655/40-25 |
| F4 | Filter | Semrock FF01-855/210-25, FF01-950/SP |
| G | Grating | LightSmyth LSFSG-1000-3212-94 |
| L1 | Achromatic Lens | Thorlabs AC508-075-B |
| L2 | Achromatic Lens | Thorlabs AC508-075-B |
| L3 | Achromatic Lens | Thorlabs AC508-075-C |
| L4 | Achromatic Lens | Thorlabs AC254-125-B |
| L5 | Achromatic Lens | Thorlabs AC508-080-B |
| L6 | Achromatic Lens | Thorlabs AC508-150-B |
| M | Broadband dielectric mirror | Thorlabs BB1-E03 |
| Obj A | Objective lens | Olympus UPLSAPO 60XW |
| Obj B | Condenser | Olympus U-AAC |
| PPLN | Periodically poled lithium niobate crystal | Covesion MSHG1550-0.5 |
| PBS | Polarizing beam splitter | Thorlabs PBS123 |
| xyGM | xy galvanometric mirror | Cambridge Technology 6220H |
| λ/2 | Half-wave plate | Thorlabs WPH05M-1030/ WPH05M-1550 |
| λ/4 | Quarter-wave plate | Thorlabs WPQ05M-1030/ WPQ05M-1550 |

| **Optoelectronic/electronic components** |
| --- |

| FD | Frequency divider | Pulse Research Lab PRL-220A |
| --- | --- | --- |
| FG | Function generator | HP 8116A |
| FIM | Fibre-coupled intensity modulator | iXblue NIR-MX-LN-10 |
| DAQ | Data acquisition card | National Instruments PCI-6110 |
| LIA | Lock-in amplifier | APE Berlin |
| PD1 | InGaAs-photodetector | Thorlabs DET01-CFC |
| PD2 | Si-photodetector | APE Berlin, a 10x10 mm active area |
| PMT | Photomultiplier tube | Hamamatsu H9565-20 |

**Table S3 Prices of the components used in the femtosecond master cavity**

| **Items** | **Supplier** | **Qty.** | **Unit Price ($)** | **Price ($)** |
| --- | --- | --- | --- | --- |
| OIM | Optizonetech | 1 pc | 220 | 220 |
| Pump | II-VI | 1 pc | 445 | 445 |
| PC | General photonics | 1 pc | 300 | 300 |
| Yb | Thorlabs | 0.3 m | 80 | 24 |
| FC | Thorlabs | 2 pcs | 215 | 430 |
| F1 | Semrock | 1 pc | 325 | 325 |
| λ/2 | Thorlabs | 1 pc | 462 | 462 |
| M | Thorlabs | 2 pcs | 77 | 154 |
| **Total:** | | | | **2360** |

1. **Principle of the passive self-synchronization**

In this section, we study the working principle of the passive self-synchronization that coherently generates two-colour laser pulses. The self-synchronization is based on the interaction between cross-phase modulation (XPM) and group velocity dispersion (GVD). The CWG cavity (**Fig. S2**) is constructed by SMFs, including both passive and active ones, that provide the XPM and GVD effects, where the copropagating laser beams at two different wavelengths ($\lambda_{1}$ and $\lambda_{2}$, i.e., 1.0 µm and 1.5 µm, respectively, in this case) interact with each other through the fibre nonlinearity, mainly considering the XPM effect in this study, although other nonlinear effects might involve in the optical evolution of individual laser beams, e.g., self-phase modulation (SPM), stimulated Raman scattering (SRS), Brillouin scattering, harmonic generation, and FWM. Under the XPM, these two optical beams can couple with each other without energy transferring^38^. The XPM process occurred in optical fibres can be well described by the coupled nonlinear Schrödinger equations (NSLs)^39^:

$\frac{\partial A_{1}}{\partial z}=-\frac{\alpha_{1}}{2}A_{1}-\beta_{11}\frac{\partial A_{1}}{\partial t}-\frac{i\beta_{21}}{2}\frac{\partial^{2}A_{1}}{\partial t^{2}}+i\gamma_{1}(\left| A_{1} \right|^{2}+2\left| A_{2} \right|^{2})A_{1}$, (1)

$\frac{\partial A_{2}}{\partial z}=-\frac{\alpha_{2}}{2}A_{2}-\beta_{12}\frac{\partial A_{2}}{\partial t}-\frac{i\beta_{22}}{2}\frac{\partial^{2}A_{2}}{\partial t^{2}}+i\gamma_{2}(\left| A_{2} \right|^{2}+2\left| A_{1} \right|^{2})A_{2}$, (2)

where $z$ is the longitudinal coordinate of the optical fibre, $A_{1}A_{2}$ denote the optical fields of pulsed laser beams at $\lambda_{1}$ and $\lambda_{2}$, $\alpha_{1}\alpha_{2}$ are the attenuation coefficient, $\beta_{11}\beta_{12}$ are related to the group velocity ($v_{gj}$, ${\beta_{1j}=1/v}_{gj}$, $j=1,2$), $\beta_{21}\beta_{22}$ are the GVD coefficients, and $\gamma_{1}\gamma_{2}$ are the nonlinear parameters.


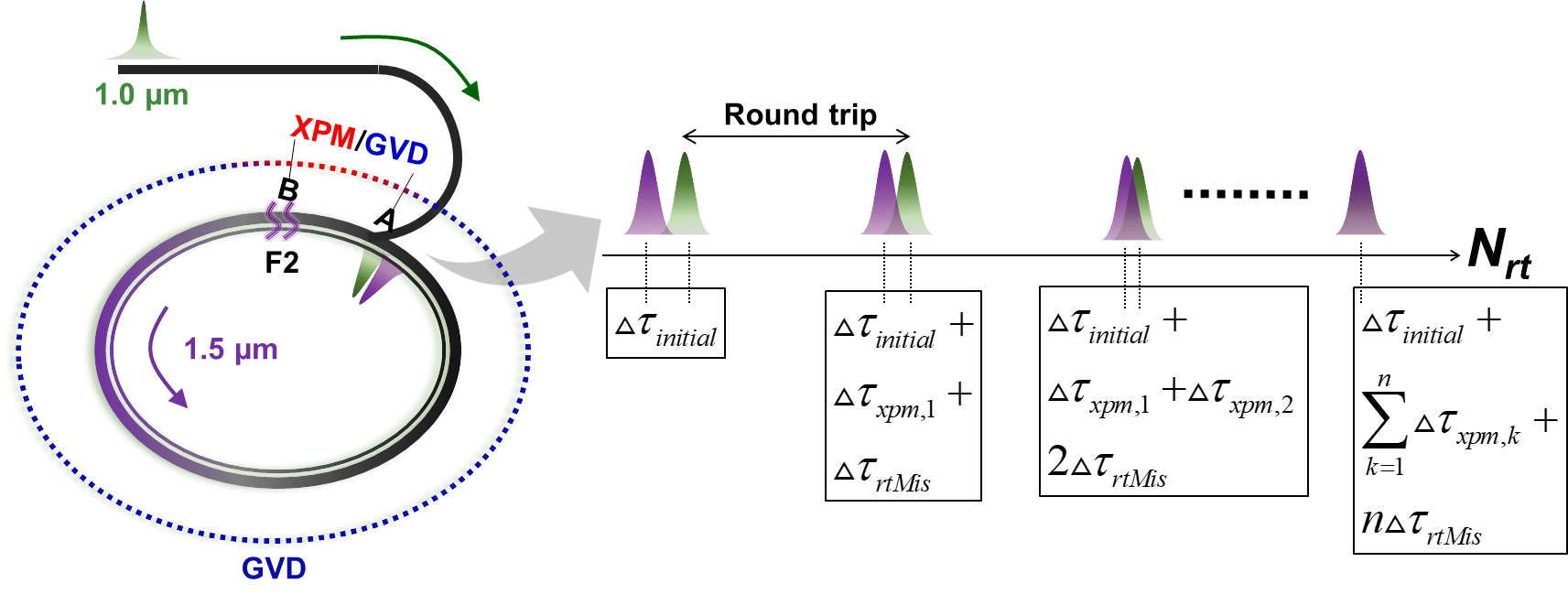


**Figure S2** | **Modelling of the passive self-synchronization in the CWG cavity**. The CWG cavity operates at 1.5 µm, where the self-stabilized pulse (via NPR effect) circulates inside the CWG cavity and collides with the injected 1.0 µm pulse every round trip in fibre section **AB**. It is noted that a portion of the optical power of the circulating 1.5 µm pulse is extracted every round trip by the fibre coupler, i.e., OIM in this case, and this attenuation is compensated by the inbuilt optical amplifier (EDFA, see **Fig. S1**). The right inset illustrates the timing evolution of these two pulses at the injection position during the self-synchronization process. Here, it is assumed that the 1.5 µm pulse is initially faster than the 1.0 µm pulse. **F2**: bandpass filter. **GVD**: group velocity dispersion. $\boldsymbol{N}_{\boldsymbol{rt}}$: the round-trip number. $\boldsymbol{\Delta}\boldsymbol{\tau}_{\boldsymbol{initial}}$: the initial time delay between two-colour pulses. $\boldsymbol{\Delta}\boldsymbol{\tau}_{\boldsymbol{xpm}}$: XPM-induced timing change.$\boldsymbol{\Delta}\boldsymbol{\tau}_{\boldsymbol{rtMis}}$: the constant round-trip time mismatch due to the FRR mismatch. **XPM**: cross-phase modulation.

The pulsed laser beams copropagating in the optical fibre have different speeds, i.e., $v_{g1}$ and $v_{g2}$. The group velocity mismatch results in an effective interaction duration, i.e., the walk-off effect, and they will finally be separated. The copropagating fibre length defined by the walk-off effect is given by $L_{c}=T_{0}/(\left| \beta_{11}-\beta_{12} \right|)$, where $T_{0}$ is the pulse width. Given that the pulse width is a few ps in this work and group velocity mismatch between the 1.0 µm and 1.5 µm laser beams is large, the copropagating fibre length is just a few centimetres. In addition to the walk-off effect, the copropagating fibre length can be further confined by the spectral filter, i.e., the bandpass optical filter (F2) placed at **B**, a certain distance from the injection location (**A**) of the 1.0 µm pulse, see **Fig. S2**.

To model the passive self-synchronization, we assume that the pulse train generated by the passively mode-locked fibre laser at 1.0 µm is stable, in terms of both intensity and FRR^31, 40-42^. Since the CWG cavity is constructed by a polarization-dependent fibre component, i.e., OIM at 1.5 µm, it can also generate a stable ultrashort pulse train from either the environmental perturbation or external injection^43-45^, the latter case in this work^46^. It should be pointed out that the cavity length of the CWG cavity has been carefully optimized to match with that of the 1.0 µm master fibre laser. From now on, we only consider the CWG cavity under the influence of the injected pulses at 1.0 µm, and the studies start from a situation that the CWG cavity has successfully produced a self-sustained pulse train, which however is not synchronized with the 1.0 µm pulse train due to the FRR mismatch.

The passive self-synchronization is initiated by the optical collision between the circulating 1.5 µm pulse and injected 1.0 µm pulse in the copropagating fibre, i.e., from **A** to **B**. During the optical collision, the XPM effect serves as an instantaneous synchronization mechanism: the optical collision between the two-colour pulses results in a frequency shift through the XPM effect. The direction of frequency shifting of the circulating pulse at 1.5 µm is determined by the timing between these two pulses, i.e., red-shift for a faster timing, or blue-shift for a slower timing (temporally referring to the injected pulse). Since the CWG cavity has a net anomalous dispersion at 1.5 µm, the circulating pulse is subsequently slowed down or speeded up according to the frequency shift. The maximum amount of the centre wavelength shifting can be estimated by:

$\Delta\lambda_{max}=\frac{{\lambda_{2}^{2}\gamma}_{2}P_{1,P}L_{c}}{\pi cT_{0}}$, (3)

where $P_{1,P}$ is the peak power of the laser pulse at $\lambda_{1}$. The corresponding maximum change of the round-trip time is then evaluated by:

$\Delta T_{max}\propto\Delta\lambda_{max}\times{GVD}_{2}\times L$, (4)

where ${GVD}_{2}$ is the group velocity dispersion at $\lambda_{2}$, and $L$ is the fibre length of the CWG cavity.

We first numerically investigate the frequency shift of the 1.5 µm pulse through the XPM effect when the two-colour pulses collide in the copropagating fibre **AB**. To this end, the coupled NSLs are solved by the well-known split-step Fourier method (SSFM)^47^, although the fourth-order Runge-Kutta in the interaction picture (RK4IP) method has also been widely applied^48,49^. It is assumed that both optical fields have a Gaussian pulse shape with a pulse width of 1.0 ps, while other key parameters used in this numerical study are given in **Table S4**. A reference frame moving with the copropagating pulse at 1.0 µm is also introduced by using a new time coordinate*,* $T=t-\beta_{11}z$, which moves at a speed of $v_{g1}$. Thus, the first-order dispersion coefficient $\beta_{12}$ of Eq. (2) is replaced by $\Delta\beta=\beta_{12}-\beta_{11}$, i.e., a coefficient regarding the mismatch of the first-order dispersion between these two colours.

**Table S4 Key parameters used in the numerical simulation**

| Pulse width, s | Wavelength, nm | $\alpha$ | $\beta_{1}$, s/m | $\beta_{2}$, s^2^/m | $\gamma$, /Wm |
| --- | --- | --- | --- | --- | --- |
| 1e^-12^ | 1020 | 0 | 4.8339e^-9^ | 2.9000e^-27^ | 3.5e^-3^ |
| 1e^-12^ | 1580 | 0 | 4.8112e^-9^ | -2.6000e^-27^ | 2.3e^-3^ |


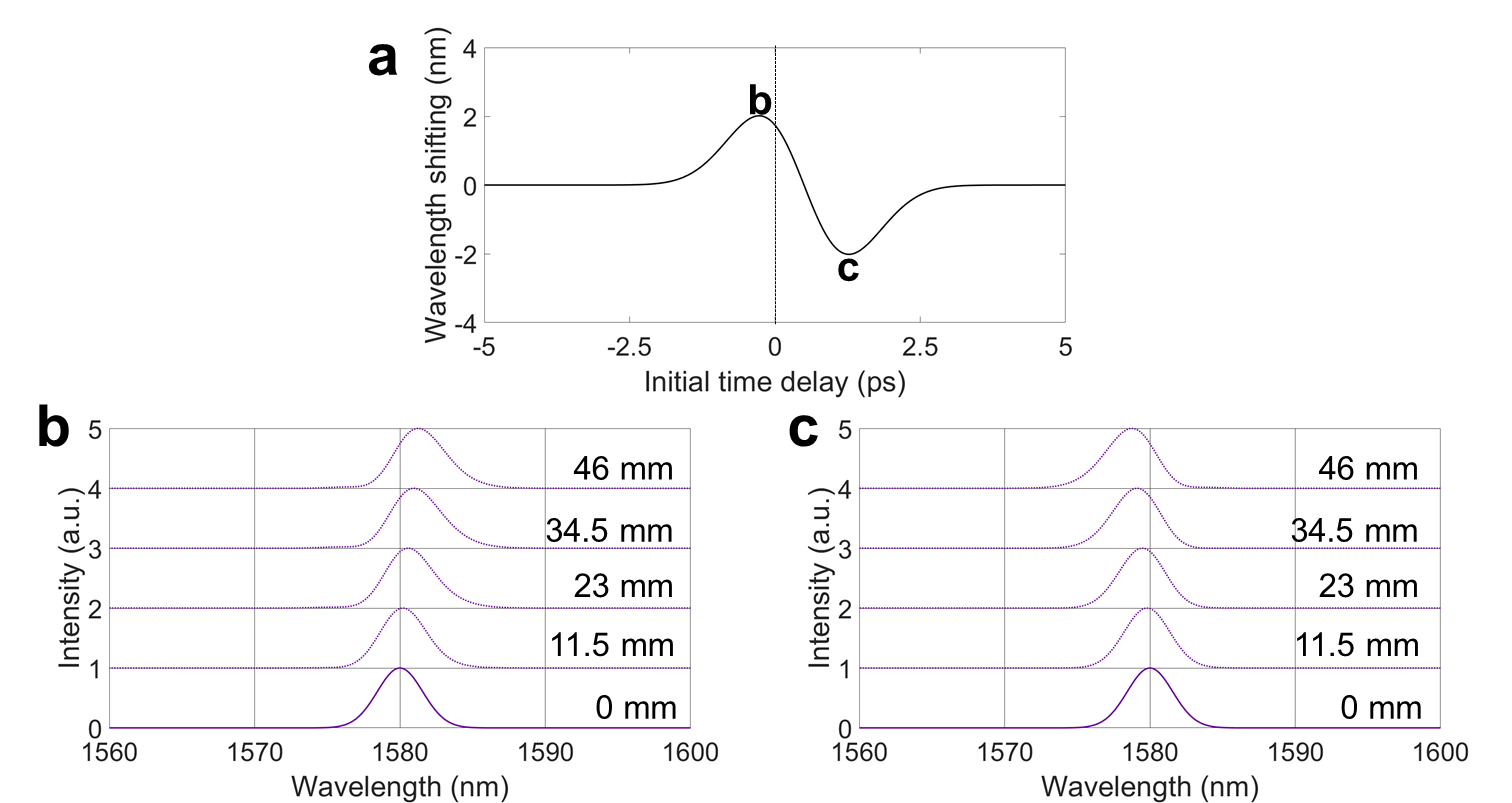


**Figure S3** | **Numerical simulation of the XPM-induced wavelength shifting**. **a.** XPM-induced wavelength shift as a function of the initial time delay between the two-colour pulses. **b,c.** Spectral evolution of the 1.5 µm pulse when it is interacting with the 1.0 µm pulse along the copropagating fibre **AB**. It is noted that the intensities have been normalized, and the spectra in **b** and **c** have been vertically offset for a better visualization.

Given that the circulating 1.5 µm pulse is stabilized by itself and the cavity loss is compensated by an intracavity optical amplifier, both loss and gain coefficients are set to zero for the simplicity. The results of the XPM-induced wavelength shifting at different timings is illustrated in **Fig. S3**. It is clear that the centre wavelength of the circulating pulse is red-shifted when it is earlier than the injected pulse. In contrast, it is blue-shifted when it is later than the injected pulse.


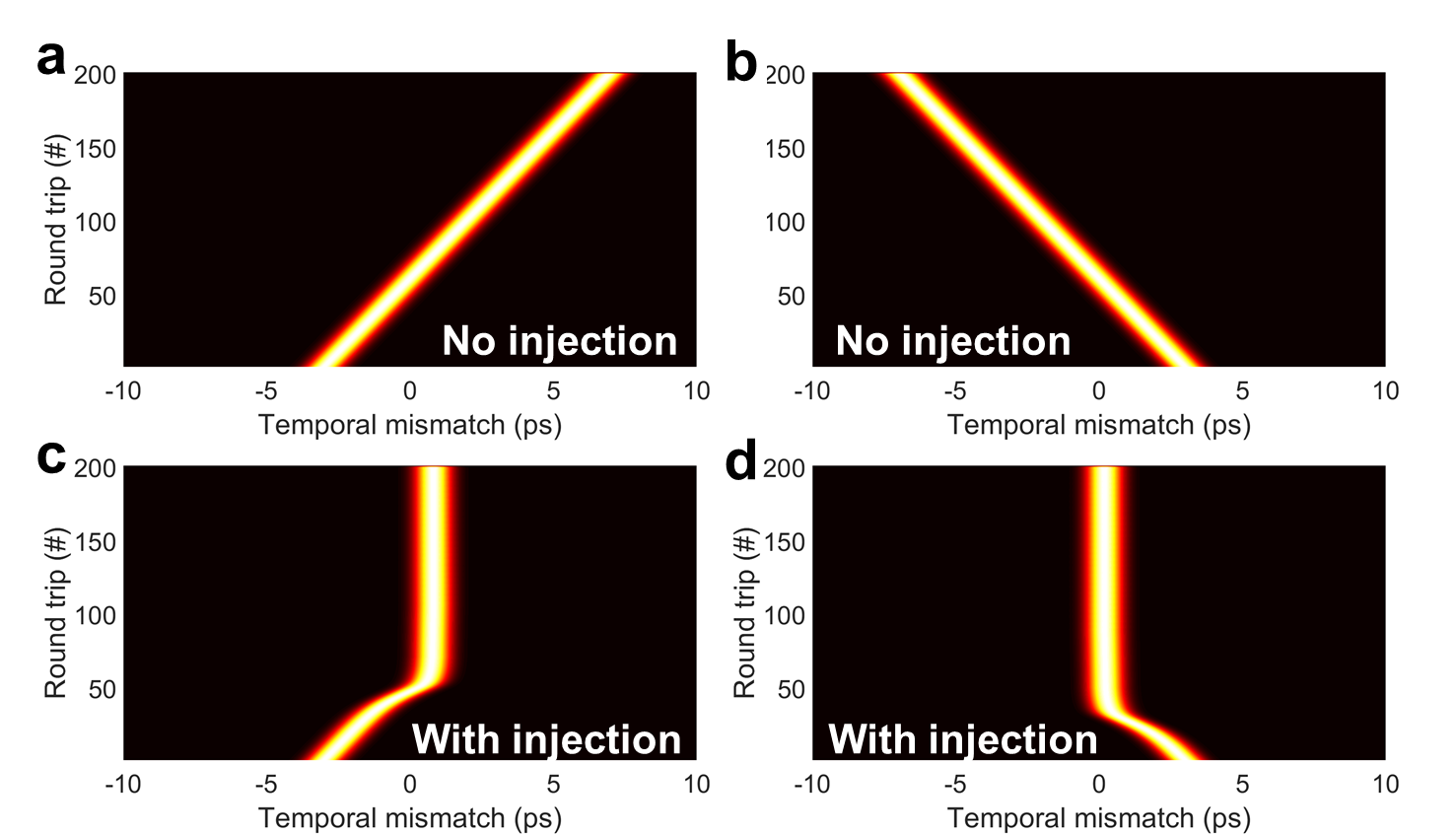


**Figure S4** | **Numerical simulation of the self-synchronization**. **a,c.** Timing evolution of the 1.5 µm pulse circulating in the CWG cavity that has a slightly lower FRR, i.e., a longer round-trip time than that of the injected pulse at 1.0 µm. **b,d.** Timing evolution for the case that the CWG cavity has a higher FRR. Here, the reference timing is 0 ps, i.e., the time when the 1.0 µm pulse is injected every round trip.

Up to now, we have only considered the pulse collision in the copropagating fibre **AB**. To give a full picture of how the circulating pulse automatically synchronizes with the injected pulses, we simulate the closed loop of the CWG cavity with an initial FRR mismatch. As shown in **Fig. S2**, the circulating pulse collides with the injected pulse in the copropagating fibre **AB** (4.6 cm in length), where both dispersion and nonlinearity are included for the simulation. Then the 1.5 µm pulse passes through a second piece of optical fibre (245.4 cm in length), where only the dispersion (GVD) effect is considered, and the propagation speed is changed according to the centre wavelength shift generated in the collision. As such, the 1.5 µm pulse circulates inside the cavity and interacts with the injected pulse every round trip in **AB**, and finally turns to a balanced state, i.e.,

$$\Delta T_{n}=\Delta\tau_{initial}+\sum_{k=1}^{n} \Delta\tau_{xpm,k}+n\Delta\tau_{rtMis}=const.$$

, (5)

$\Delta\tau_{initial}$ is the initial time delay between the two-colour pulses at the beginning of the simulation, $\Delta\tau_{xpm,k}$ is the XPM-induced timing change at round trip $k$, and $\Delta\tau_{rtMis}$ is the constant mismatch of the round-trip time due to the FRR mismatch.

The simulation starts from an initial timing delay of 3 ps and a round-trip time mismatch of 50 fs. The results are shown in **Fig. S4**. Even with a large timing delay at the beginning, the circulating pulse of the CWG cavity can self-adjust to synchronize with the 1.0 µm pulse train within less than 50 round trips, i.e., about 625 ns.

This self-adjustable synchronization and mode-locking mechanism ensures excellent RIN performance and low timing jitter of both pump and Stokes beams. In addition, the laser source exhibits good long-term power stability which arises from the natural characteristics of fibre lasers, particularly: 1) a high surface-to-volume ratio (avoiding excessive heating) and the guiding effect of optical fibres, which avoids thermo-optical problems even under conditions of significant heating; 2) the flexibility of optical fibres enable being coiled, and the lightwave propagating in optical fibres is well shielded from the environment (e.g., the turbulence from air or dust), which make fibre lasers compact and reliable.

1. **Versatilities of the XPM-based self-synchronization in generating two-colour multiple pulses**

Another specialty of this two-colour pulsed fibre laser is that it can generate synchronized two-colour multiple pulses. Since the laser beam of the CWG cavity is “modulated” by the pulses injected from the master laser through the XPM effect, and the modulation pattern follows the pulse pattern of the injection, this initial modulation pattern can gradually evolve into the same short pulse pattern through the pulse compression capability and synchronize with the injected pattern^46^. Here, we manipulate the passively mode-locked cavity at 1.0 µm to generate different patterns of multiple pulses through the peak power clamping mechanism^50^. The synchronized two-colour patterns with 1, 2 and 4 pulses in a single round trip are shown in **Fig. S5**.


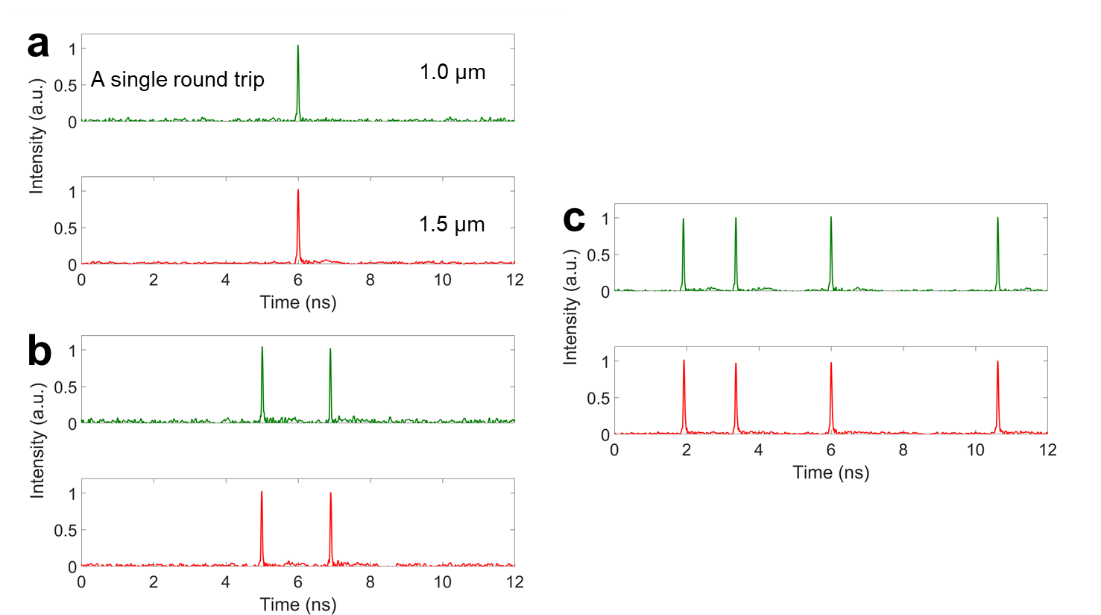


**Figure S5** | **Synchronized two-colour multiple pulses**. **a**. Typical synchronized two-colour pulse, containing only a single pulse in each round trip. **b**. Two pulses in each round trip. **c**. Four pulses in each round trip.

1. **Additional characterizing data of the two-colour pulsed fibre laser**


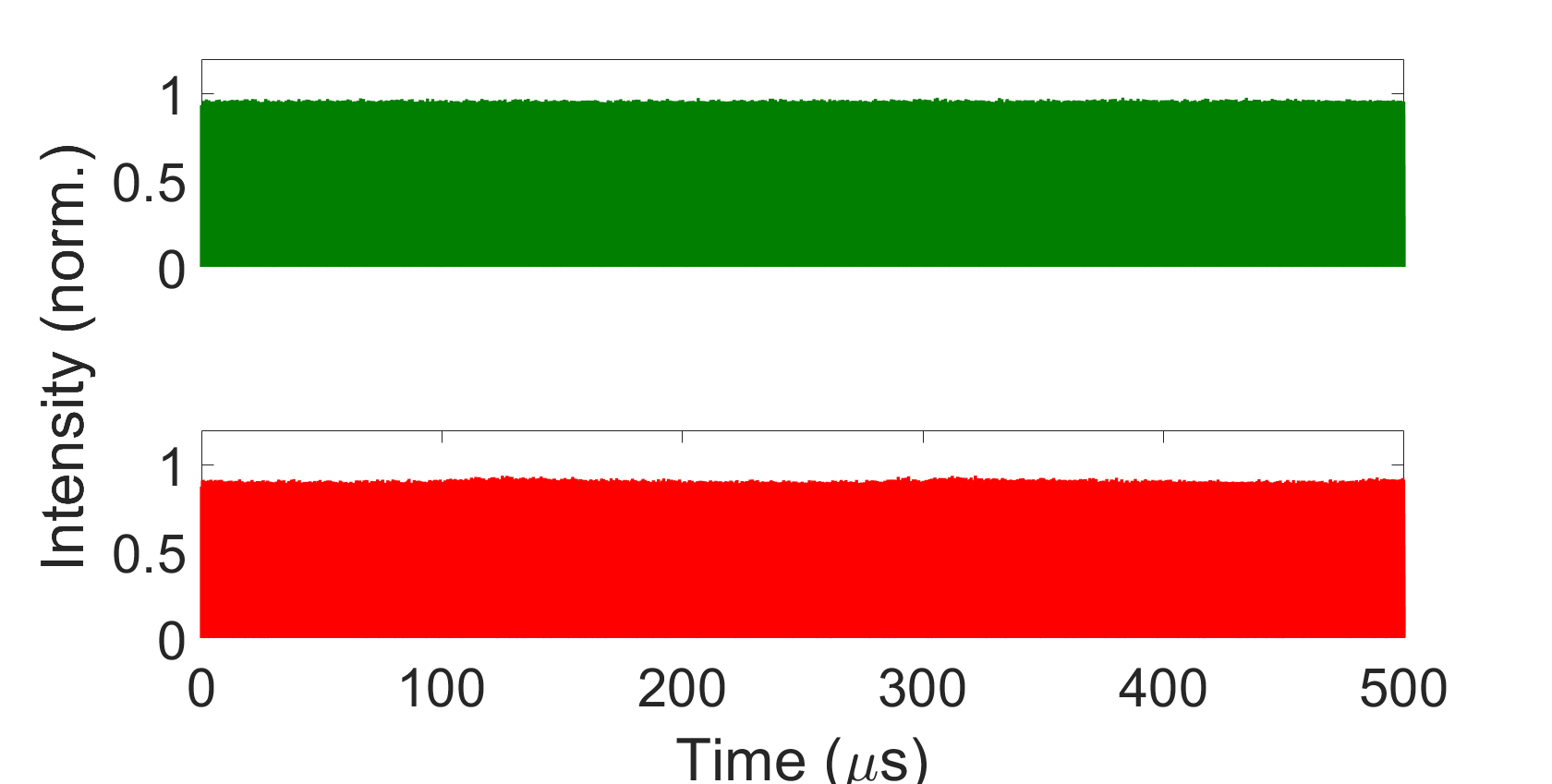


**Figure S6** | **Extended pulse trains (up to 500 µs) of the Stokes and pump laser beams.**


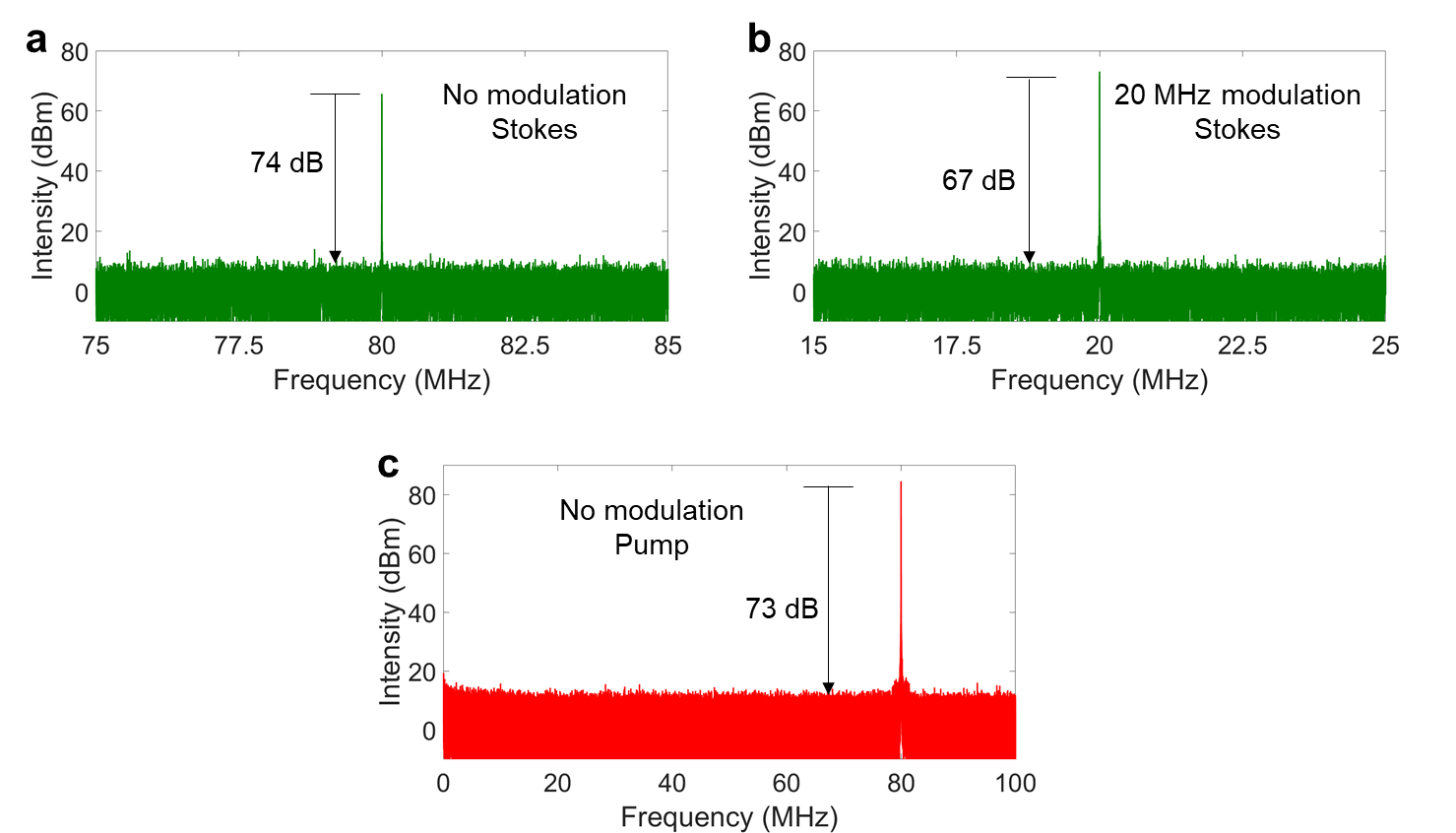


**Figure S7** | **Radio frequency (RF) spectra of the two-colour pulsed fibre laser. a**. RF spectrum of the Stokes beam that has an FRR of 80 MHz, at which frequency it shows a signal-to-noise ratio (SNR) of 74 dB. **b**. RF spectrum of the Stokes beam modulated at 20 MHz, i.e., the modulation frequency used for SRS imaging. **c**. RF spectrum of the pump beam, which shows a SNR of 73 dB at 80 MHz.

1. **Relative intensity noise (RIN) measurement**

In this section, we evaluate the relative intensity noise (RIN) of our two-colour fibre laser, and compare it with a commonly-used SC-based two-colour pulsed fibre laser, as well as a Ti:sapphire fs laser that has a RIN approaching the shot noise limit. To this end, a SC-based two-colour pulsed fibre laser is built by using a configuration similar to that of Ref. 23. It starts from a commercial fs fibre laser, Alnair Labs EYE-1000C, which has an FRR of 45 MHz. Its output is first amplified to about 500 mW and is then used to pump a short piece of HNF (OFS HNLF-SPINE ZDW 1540, 12 cm in length). The dispersion of this HNF has been optimized for a stable phase match and high nonlinear coefficient. Its zero-dispersion wavelength (ZDW, around 1566 nm) has also been stabilized along the fibre. **Fig. S8a** shows the generated SC spectrum after the HNF. As can be observed, the newly-generated frequency components range from ~1000 to >1700 nm. It is noted that the measurement of longer wavelengths beyond 1700 nm is limited by the operating wavelength range of the optical spectrum analyser (600 – 1700 nm). Then, the blue-shifted wavelength complements in the 1.0 µm region are filtered out by a narrowband tunable filter that can cover a wavelength range of ~1010 – 1100 nm, as shown in **Fig. S8b**. To study the intensity stability of the SC pulses, the real-time pulse trains of the SC pump (**Figs. S8c** and **d**) and blue-shifted SC at around 1060 nm (**Figs. S8e–h**) is recorded, and plotted in two different time spans, i.e., 200 µs and 1 µs, respectively. The results show that there is a large degradation of the intensity stability in the 1.0 µm region, compared to that of the SC pump. This is because that the generation of new frequencies is usually initiated by the noise in the SC generation^29^, particularly when it is pumped by long pulses, in which case the spectral broadening is mainly dominated by the FWM, modulation instability (MI) and Raman scattering. The intensity fluctuation can produce a large pulse-to-pulse variation and thus a poor temporal coherence^30^. It is worthy to note that, many other methods have consequently been proposed to improve the stability of the SC generation, such as initiating with a pulse-seed^51^, introducing a feedback loop^52^, modulating the pump pulse train^53^, and tapering the photonics crystal fibre^54,55^.


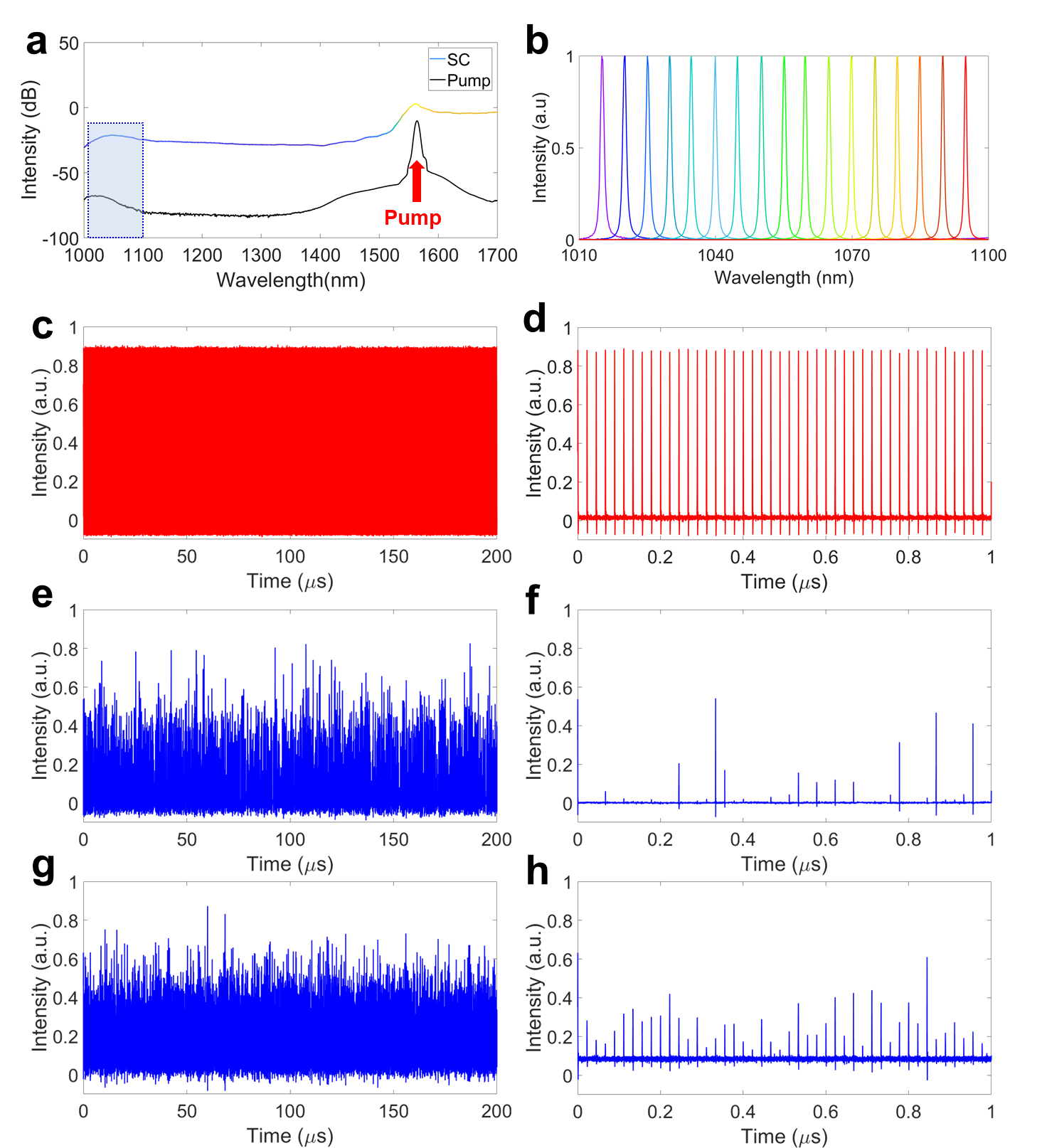


**Figure S8** | **Spectral and temporal performances of a SC-based two-colour pulsed fibre laser**. **a**. Optical spectrum of the SC generation pumped by a commercial grade fs fibre laser at 1.5 µm (black curve, before being amplified). **b**. Spectral tuning in the blue-shifted region (1010 – 1100 nm, the blue dotted rectangle in **a**), by using a narrowband tunable filter. **c,d**. Real-time pulse trains of the 1.5 µm pump laser, shown in two different time spans, i.e., 200 µs and 1 µs, respectively. **e.f.** Real-time pulse trains of the blue-shifted wavelength at around 1.0 µm, filtered out by a 10 nm bandpass filter, i.e., 1050 – 1060 nm. **g,h**. Real-time pulse trains of the blue-shifted wavelength at around 1.0 µm, filtered out by a 100 nm bandpass filter, i.e., 1000 – 1100 nm. All pulse trains were recorded by a 20 GHz real-time oscilloscope.

1. **Videos and Tables**

The videos show z-scans of U2OS cells (both CARS and SRS, **Videos S1** and **S2**, respectively) and mouse superior vena cava tissue cryo-section (both CARS and SRS, **Videos S3** and **S4**, respectively). Here, we utilize the z stage to move the focus spot through the sample at a step size of 1 µm for the cell samples and 4 µm in the case of the mouse tissue. The pump and Stokes focal powers were set to 33 mW and 66 mW, respectively, for both samples. The pixel dwell time and the integration time of the lock-in amplifier were 6.4 µs and 2 µs, respectively. In the case of the cells, we increased the pixel dwell time to 12.5 µs and kept the integration time constant.

**Video S1: Z-scanned CARS imaging of U2OS cells**

**Video S2: Z-scanned SRS imaging of U2OS cells**

**Video S3: Z-scanned CARS imaging of mouse superior vena cava tissue cryo-section**

**Video S4: Z-scanned SRS imaging of mouse superior vena cava tissue cryo-section**

**Table S5. Pixel sizes and total acquisition times of the images**

| **Figure** | **Pixel size** | **Pixel dwell time** | **Total acquisition time** |
| --- | --- | --- | --- |
| **4a and b** | 512x512 | 12.5 µs | 3.27 s |
| **4c and d** | 256x256 (2x zoom) | 51.2 µs | 3.35 s |
| **5b and c** | 512x512 for single image, 9 images were utilised for stitching | 6.4 µs | 15.1 s  (+5.0 s sample movement) |
| **5d and e** | 512x512 for single image, 19 images were utilised for stitching | 6.4 µs | 31.8 s  (+10 s sample movement) |
| **6a** | 512x512 | 1.6 µs | 0.42 s |
| **6b and c** | 512x512 | 3.2 µs | 0.84 s |
| **6d** | 512x512 for single image, 145 images in volumetric assembly | 3.2 µs | 121 s  (+29 s sample movement) |
| **6e** | 512x512 | 3.2 µs | 0.84 s |

**References**

1. Ozeki, Y. et al. Stimulated Raman scattering microscope with shot noise limited sensitivity using subharmonically synchronized laser pulses. Opt. Express **18**, 13708–13719 (2010).
2. Wang, K. et al. Synchronized time-lens source for coherent Raman scattering microscopy. Opt. Express **23**, 24019–24024 (2010).
3. Ozeki, Y. et al. Stimulated Raman hyperspectral imaging based on spectral filtering of broadband fibre laser pulses. Opt. Lett. **37**, 431–433 (2011).
4. Wang, K. et al. Time-lens based hyperspectral stimulated Raman scattering imaging and quantitative spectral analysis. J. Biophotonics **6**, 815–820 (2013).
5. Nose, K. et al. Sensitivity enhancement of fiber-laser-based stimulated Raman scattering microscopy by collinear balanced detection technique. Opt. Express **20**, 13958–13965 (2012).
6. Karpf, S., Eibl, M., Wieser, W., Klein, T. & Huber, R. A time-encoded technique for fibre-based hyperspectral broadband stimulated Raman microscopy. Nat. Commun. **6**, 6784 (2015).
7. Zhai, Y. H. et al. Multimodal coherent anti-Stokes Raman spectroscopic imaging with a fiber optical parametric oscillator. Appl. Phys. Lett. **98**, 191106 (2011).
8. Baumgartl, M. et al. All-fiber laser source for CARS microscopy based on fiber optical parametric frequency conversion. Opt. Express **20**, 4484–4493 (2012).
9. Lefrancois, S. et al. Fiber four-wave mixing source for coherent anti-Stokes Raman scattering microscopy. Opt. Lett. **37**, 1652–1654 (2012).
10. Yang, K. et al. Low-repetition-rate all-fibre integrated optical parametric oscillator for coherent anti-Stokes Raman spectroscopy. Opt. Express **26**, 17519–17528 (2018).
11. Baumgartl, M. et al. Alignment-free, all-spliced fibre laser source for CARS microscopy based on four-wave-mixing. Opt. Express **20**, 21010–21018 (2012).
12. Chemnitz, M. et al. Widely tuneable fibre optical parametric amplifier for coherent anti-Stokes Raman scattering microscopy. Opt. Express **20**, 26583–26595 (2012).
13. Lamb, E. S. et al. Fibre optical parametric oscillator for coherent anti-Stokes Raman scattering microscopy. Opt. Lett. **38**, 4154–4157 (2013).
14. Gottschall, T. et al. Fibre-based optical parametric oscillator for high resolution coherent anti-Stokes Raman scattering (CARS) microscopy. Opt. Express **22**, 21921–21928 (2014).
15. Andresen, E. R., Nielsen, C. K., Thøgersen, J. & Keiding, S. R. Fibre laser-based light source for coherent anti-Stokes Raman scattering microspectroscopy. Opt. Express **15**, 4848–4856 (2007).
16. Pegoraro, A. F. et al. All-fibre CARS microscopy of live cells. Opt. Express **17**, 20700–20706 (2009).
17. Krauss, G. et al. Compact coherent anti-Stokes Raman scattering microscope based on a picosecond two-color Er:fibre laser system. Opt. Lett. **34**, 2847–2849 (2009).
18. Andresen, E. R., Berto, P. & Rigneault, H. Stimulated Raman scattering microscopy by spectral focusing and fibre-generated soliton as Stokes pulse. Opt. Lett. **36**, 2387–2389 (2011).
19. Xie, R. et al. Multi-modal label-free imaging based on a femtosecond fibre laser. Biomed. Opt. Express **5**, 2390–2396 (2014).
20. Crisafi, F. et al. Multimodal nonlinear microscope based on a compact fiber-format laser source. Spectrochim Acta A Mol. Biomol. Spectrosc. **188**, 135–140 (2018).
21. Gambetta, A. et al. Fibre-format stimulated-Raman-scattering microscopy from a single laser oscillator. Opt. Lett. **35**, 226–228 (2010).
22. Selm, R. et al. Ultrabroadband background-free coherent anti-Stokes Raman scattering microscopy based on a compact Er:fibre laser system. Opt. Lett. **35**, 3282–3284 (2010).
23. Freudiger, C. W. et al. Stimulated Raman scattering microscopy with a robust fibre laser source. Nat. Photon. **8**, 153–159 (2014).
24. Chen, K., Wu, T., Zhou, T., Wei, H. & Li, Y. Cascaded Dual-Soliton Pulse Stokes for Broadband Coherent Anti-Stokes Raman Spectroscopy. IEEE Photonics J. **8**, 1–8 (2016).
25. Tu, H. et al. Stain-free histopathology by programmable supercontinuum pulses. Nat. Photon. **10**, 534–540 (2016).
26. Orringer, D. A. et al. Rapid intraoperative histology of unprocessed surgical specimens via fibre-laser-based stimulated Raman scattering microscopy. Nat. Biomed. Eng. **1**, 0027 (2017).
27. Coluccelli, N. et al. Er/Tm:fibre laser system for coherent Raman microscopy. Opt. Lett. **39**, 3090–3093 (2014).
28. Riek, C. et al. Stimulated Raman scattering microscopy by Nyquist modulation of a two-branch ultrafast fibre source. Opt. Lett. **41**, 3731–3734 (2016).
29. Dudley, J. M., Genty, G. & Coen, S. Supercontinuum generation in photonic crystal fibre. Rev. Mod. Phys. **78**, 1135–1184 (2006).
30. Mussot, A. et al. Spectral broadening of a partially coherent CW laser beam in single-mode optical fibres. Opt. Express **12**, 2838–2843 (2004).
31. Chong, A., Buckley, J., Renninger, W. & Wise, F. All-normal-dispersion femtosecond fibre laser. Opt. Express **14**, 10095–10100 (2006).
32. Strickland, D. & Mourou, G. Compression of amplified chirped optical pulses. Opt. Commun. **56**, 219–221 (1985).
33. <https://www.thorlabs.com/newgrouppage9.cfm?objectgroup_id=2729>
34. Wei, X. et al. Ultrafast time-stretch imaging at 932 nm through a new highly-dispersive fibre. Biomed. Opt. Express **7**, 5208–5217 (2016).
35. Broderick, N. G. R. et al. Optical pulse compression in fibre Bragg gratings. Phys. Rev. Lett. **79**, 4566, (1997).
36. Lenz, G., Eggleton, B. J. & Litchinitser, N. Pulse compression using fibre gratings as highly dispersive nonlinear elements. J. Opt. Soc. Am. B **15**, 715–721 (1998).
37. https://agiltron.com/product/motorized-fibre-optic-tunable-filter/
38. Agrawal, G. P. Nonlinear Fibre Optics, 5th ed. (Academic, 2012).
39. Schadt, D. & Jaskorzynska, B. Generation of short pulses from CW light by influence of crossphase modulation (CPM) in optical fibres. Electron. Lett. **23**, 1090–1091 (1987).
40. Haus, H. A. Theory of mode locking with a fast saturable absorber. J. Appl. Phys. **46**, 3049 (1975).
41. Ippen, E. P. Principles of passive mode locking. Appl. Phys. B **58**, 159 (1994).
42. Tamura, K., Ippen, E. P., Haus, H. A. & Nelson, L. E. 77-fs pulse generation from a stretched-pulse mode-locked all-fibre ring laser. Opt. Lett. **18**, 1080–1082 (1993).
43. Krausz, F. et al. Self-starting additive-pulse mode locking of a Nd:glass laser. Opt. Lett. **15**, 1082–1084 (1990)
44. Tamura, K., Haus, H. A. & Ippen, E. P. Self-starting additive pulse modelocked erbium fibre ring laser. Electron. Lett. **28**, 2226 (1992).
45. Haus, H. A., Ippen, E. P. & Tamura, K. Additive-pulse modelocking in fibre lasers. IEEE J. Quantum Electron. **30**, 200 (1994).
46. Wei, X. et al. Addressing a cavity with patterns at ultra-wideband detune. in CLEO: 2015, OSA Technical Digest (online) (Optical Society of America, 2015), paper SW4L.6. G. P. Agrawal, Nonlinear Fibre Optics, 5th ed. (Academic, 2012).
47. Fisher, R. A. & Bischel, W. K. The role of linear dispersion in plane‐wave self‐phase modulation. Appl. Phys. Lett. **23**, 661 (1973).
48. Hult, J. A Fourth-Order Runge–Kutta in the Interaction Picture Method for Simulating Supercontinuum Generation in Optical Fibres. J. Lightwave Technol. **25**, 3770–3775 (2007).
49. Wu, S., Hsiang, W. & Lai, Y. Laser dynamics and relative timing jitter analysis of passively synchronized Er- and Yb-doped mode-locked fibre lasers. J. Opt. Soc. Am. B **31**, 1508–1515 (2014).
50. Tang, D. Y., Zhao, L. M., Zhao, B. & Liu, A. Q. Mechanism of multisoliton formation and soliton energy quantization in passively mode-locked fibre lasers. Phys. Rev. A **72**, 043816 (2005).
51. Solli, D. R., Ropers, C. & Jalali, B. Active control of rogue waves for stimulated supercontinuum generation. Phys. Rev. Lett. **101**, 233902 (2008).
52. Moselund, P. M., Frosz, M. H., Thomsen, C. L. & Bang, O. Back-seeding of higher order gain processes in picosecond supercontinuum generation. Opt. Exp. **16**, 11954–11968 (2008).
53. Genty, G., Dudley, J. M. & Eggleton, B. Modulation control and spectral shaping of optical fibre supercontinuum generation in the picosecond regime. Appl. Phys. B **94**, 187–194 (2009).
54. Kudlinski, A. et al. Control of pulse-to-pulse fluctuations in visible supercontinuum. Opt. Exp. **18**, 27445–27454 (2010).
55. Møller, U. et al. Power dependence of supercontinuum noise in uniform and tapered PCFs. Opt. Exp. **20**, 2851–2857 (2012).
